# Bintrafusp alfa for patients with recurrent or metastatic olfactory neuroblastoma

**DOI:** 10.1007/s00262-026-04462-4

**Published:** 2026-06-17

**Authors:** Nyall R. London, Dara Bracken-Clarke, Elisabetta Xue, Isaac Brownell, Wojciech K. Mydlarz, Evrim B. Turkbey, Lisa M. Cordes, Jennifer L. Marté, Lisa M. Rooper, Shrey B. Shah, Michell Manu, Zoukaa B. Sargi, James L. Gulley, Charalampos S. Floudas

**Affiliations:** 1 Sinonasal and Skull Base Tumor Section, Surgical Oncology Program, Center for Cancer Research, National Cancer Institute, National Institutes of Health, Bethesda, MD USA; 2 Department of Otolaryngology–Head and Neck Surgery, Johns Hopkins University School of Medicine, Baltimore, MD USA; 3 Center for Immuno-Oncology, Center for Cancer Research, National Cancer Institute, National Institutes of Health, Bethesda, MD USA; 4 Dermatology Branch, National Institute of Arthritis and Musculoskeletal and Skin Diseases, National Institutes of Health, Bethesda, MD USA; 5 Radiology and Imaging Sciences, Clinical Center, National Institutes of Health, Bethesda, MD USA; 6 Department of Pathology, Johns Hopkins University School of Medicine, Baltimore, MD USA; 7 Department of Otolaryngology–Head and Neck Surgery, Rutgers New Jersey Medical School, Newark, NJ USA; 8 Clinical Research Directorate, Frederick National Laboratory for Cancer Research, Frederick, MD USA; 9 Department of Otolaryngology–Head and Neck Surgery, Sylvester Comprehensive Cancer Center, University of Miami Miller School of Medicine, Miami, FL USA

**Keywords:** Olfactory neuroblastoma, Immune checkpoint blockade, TGF-beta inhibition, Rare tumors, Trismus, ^68^Ga-DOTATATE PET/CT

## Abstract

**Background:**

There is no established treatment for patients with recurrent/metastatic (R/M) olfactory neuroblastoma (ONB), a rare sinonasal tumor. We conducted the first immunotherapy clinical trial (NCT05012098) in patients with R/M ONB, to evaluate the activity of the bifunctional TGF-β “trap”/anti-PD-L1 fusion protein bintrafusp alfa (BA).

**Methods:**

Patients received BA (1200 mg, intravenously) every 2 weeks. Primary endpoint was overall response rate (ORR); secondary objectives included safety, progression-free survival (PFS), and overall survival (OS).

**Results:**

Between June 2022 and June 2023, 11 patients received treatment (Cohort 1, no prior immunotherapy, n=9; Cohort 2, prior immunotherapy received, n=2); enrollment was stopped due to BA development discontinuation. No objective responses were observed. In Cohort 1, 4 patients (of 8 evaluable, 50%) had stable disease (SD), three confirmed and long lasting; median PFS was 3.7 months, median OS was not reached; and one of SD patients had a complete metabolic response on ^68^Ga-DOTATATE imaging. In Cohort 2, 1 patient had SD; median PFS was 3 months, and median OS was 6.8 months. Grade 3/4 treatment-related adverse events occurred in 3 patients (immune-mediated diabetes, esophageal hemorrhage, anemia, epistaxis).

**Conclusions:**

Although the primary endpoint was not met, BA showed a manageable safety profile and a subset of patients derived clinical benefit with prolonged stable disease, including one patient with a complete metabolic response. This first clinical trial of systemic therapy in R/M ONB serves as proof of clinical trials feasibility in this setting and supports further investigation of combinatorial immunotherapy strategies in this patient population.

*Trial registration* ClinicalTrials.gov Identifier: NCT05012098, https://clinicaltrials.gov/ct2/show/NCT05012098. Registered 18 August 2021.

**Supplementary Information:**

The online version contains supplementary material available at https://doi.org/10.1007/s00262-026-04462-4.

## Background

Olfactory neuroblastoma (ONB), also known as esthesioneuroblastoma, is a malignant neoplasm of the nasal vault, arising from the olfactory neuroepithelium [1]. It is a very rare tumor (0.4 cases per million people), comprising up to 6% of all sinonasal tumors, and it affects men and women equally [2]. The origin of ONB is neuroectodermal and histologically it expresses neuroendocrine markers, as well as SSTR2. Histologic evaluation includes the four-tiered Hyams grading system [3], which is prognostically important [2]. ONB tumors are characterized by a sparse mutational landscape with low mutation burden and microsatellite stable status; recurrent somatic mutations have been reported in *TP53*, *SMARCA4*, *IDH2*, *PIK3CA*, *NF1*, *CDKN2A*, *DMD*, *CTNNB1*, and other genes [4, 5].

Prognosis is favorable, with 5-year overall survival of 70–90% [6] but recurrence is up to 30% [7] and predominantly local, but distant metastases confer poor prognosis, esp. visceral (pulmonary, hepatic) vs. osseous and spinal cord drop metastases [8]. Clinical staging can follow the Kadish-Morita, Dulguerov, or American Joint Committee on Cancer systems, of which the Kadish system is the most widely accepted (stage KM-A: within nasal cavity, stage KM-B: extension to paranasal sinuses, stage KM-C: extension beyond paranasal sinuses, stage KM-D: cervical lymph nodes) [9]. A recent large retrospective analysis of an international cohort by the International Network for Sinonasal Cancers and Skull Base Tumours (INSICA) evaluating predictive utility for survival outcomes proposed the Kadish-INSICA system, consolidating the KM stages A and B and stratifying KM group C per dural infiltration (stage KI-A: within nasal cavity/paranasal sinuses; KI-B: beyond sinuses, no dural infiltration; KI-C: beyond sinuses, dural infiltration; KI-DN: neck nodal metastases; and KI-DM: distant metastases) [10].

Primary treatment consists of surgical resection and adjuvant radiotherapy [9], with induction chemotherapy in selected patients with high Hyams grade or advanced locoregional disease [11]. Because of the rarity of ONB, until now, there has been no prospective clinical study of systemic treatments for metastatic or recurrent ONB. Current guidelines list chemotherapy combinations (cisplatin or carboplatin plus etoposide, or cyclophosphamide plus doxorubicin plus vincristine) for high-grade ONB [12] based on small retrospective series. A systematic review of the management strategies for metastatic ONB reported the use of 21 different chemotherapy regimens, most commonly platinum-based (66%), with etoposide part of a combination in 37% [8]. Sporadic cases of activity of various agents were reported in the literature in the 2010s: biological agents such as imatinib [13], sunitinib with/without cetuximab [14, 15], everolimus with/without cisplatin [16, 17], bevacizumab [18], vismodegib [17], pazopanib with docetaxel [17], and SSTR2-targeted peptide receptor radionuclide therapy (PRRT; ^111^In-octreotide, ^177^Lu-DOTATATE) [19]; the only published report of immunotherapy in ONB was in the context of a rare tumor basket clinical trial of combined immune checkpoint blockade (nivolumab with ipilimumab) inducing a response in an patient with metastatic ONB (final results of this trial not published yet) [20].

Immune checkpoint blockade (ICB) has shown activity in many cancers, but responses are limited to a minority of patients. In advanced cancers, transforming growth factor β (TGF-β) suppresses antitumor immune responses and drives tumor angiogenesis and epithelial-to-mesenchymal transition, favoring tumor progression. Bintrafusp alfa (BA) is a bifunctional TGF-β “trap”/anti-PD-L1 fusion protein composed of the extracellular domain of the TGF-βRII fused to a human IgG1 antibody blocking PD-L1. Phase 1 and 2 trials of BA by our group showed a manageable safety profile with responses in 30.5% and in 10% of patients with ICB-naive and ICB-resistant HPV-associated tumors, respectively[21], and additional trials showed promising clinical activity with a manageable safety profile in a HPV-associated [22–24] and other cancers [25–27], with tumor responses occurring in PD-L1 high but also low/negative tumors [21, 27].

Initial results of PD-L1 immunohistochemistry in ONB were negative [28], but subsequent results reported PD-L1-positive ONB tumors, primary and metastatic [29, 30], associated with higher CD8 lymphocytes in the tumor and stroma [29]. Additionally a subset of ONBs with higher CD8 infiltration was shown to have higher expression of *TGFB* [30], which may drive immunosuppression in the microenvironment, and TGF-β signaling pathway genes were enriched in patients experiencing ONB recurrence following curative treatment [31]. These preclinical findings suggest that concurrent blockade of immune checkpoint PD-L1 and the TGF-β immunosuppressive pathway with BA is a rational therapeutic strategy for ONB.

We report here the results of the first clinical trial conducted for ONB, a phase 2 nonrandomized clinical trial designed to examine the clinical activity of the combination of BA in patients with recurrent or metastatic ONB.

## Methods

### Study design and participants

This is a phase 2 single-arm clinical trial (NCT05012098) conducted at the Center for Cancer Research of the National Cancer Institute (NCI), approved by the National Institutes of Health (NIH) Institutional Review Board (IRB). The trial was conducted in accordance with the Declaration of Helsinki and Good Clinical Practice standards. Participants provided written informed consent. Eligible patients were 18 years or older and had histologically confirmed recurrent or metastatic (R/M) ONB, measurable lesions per Response Evaluation Criteria in Solid Tumors (RECIST; version 1.1), Eastern Cooperative Oncology Group (ECOG) Performance Status (PS) of 0, 1, or 2, adequate organ function as defined in the protocol, and at least 1 prior systemic therapy including a platinum agent (complete eligibility criteria in Supplementary Material). Patients without prior ICB were enrolled to Cohort 1 (ICB-naïve, ICN), while patients with prior ICB exposure were enrolled to Cohort 2 (ICB-resistant, ICR).

### Procedures

Participants in both cohorts received the same treatment, consisting of intravenous BA, 1200 mg, every 2 weeks. Treatment continued until disease progression, unacceptable toxicity (as defined in the protocol), treatment withdrawal, intercurrent illness preventing further administration, or completion of 1-year treatment (treatment could continue beyond 1 year). Dose reductions were allowed (600 mg, 300 mg); interruptions were allowed for the management of adverse events (AEs). Imaging computed tomography (CT) or magnetic resonance imaging (MRI) for tumor response assessment by RECIST 1.1 was performed every 8 weeks. Blood samples for correlative research were drawn at baseline and at defined timepoints per protocol.

### Outcomes

The primary objective was to determine the clinical activity of BA in patients with R/M ONB that are immune checkpoint blockade-naïve (Cohort 1), by assessment of objective response rate (ORR, defined as the proportion of patients with confirmed complete response (CR) or partial response (PR) by RECIST 1.1). Secondary endpoints included toxicity (characterized and graded by NCI Common Terminology Criteria for Adverse Events (CTCAE) version 5) of BA in patients with R/M ONB; duration of response (DOR, time from meeting CR or PR until date of disease progression or death from any cause), progression-free survival (PFS, time from first study treatment to date of disease progression or death from any cause), and overall survival (OS, time from first study treatment to death from any cause). Exploratory endpoints included the evaluation of ORR in patients with ICR R/M ONB (Cohort 2) and of peripheral blood immune cell populations, cytokines, and soluble proteins throughout treatment. Cohort 1 and Cohort 2 will be reported separately for the secondary and exploratory endpoints.

### Statistical analysis

Cohort 1 followed a Simon two-stage design to enroll 12 evaluable participants in the first stage and if 1 or more responses were observed, to continue to a total of 21 participants, considering an outcome of 3 or more of 21 responses (14.3%) sufficiently interesting to warrant further study. Cohort 2 was designed to enroll up to a total of 8 evaluable patients with ICR R/M ONB. Enrollment to this cohort was parallel to Cohort 1. All patients who received at least one dose of study drug were considered for safety assessment. All patients who received at least one dose of any study drug and had disease re-evaluation or exhibited objective disease progression were considered for efficacy assessment.

Descriptive statistics were used to summarize the study results. OS PFS were estimated using the Kaplan–Meier method. Statistical analyses were performed using R version 4.3.3 (R Core Team).

## Results

### Patient characteristics

The study opened to accrual in April 2022. Between June 2022 and June 2023, 11 patients with R/M ONB were screened, enrolled, and received treatment. In January 2024, with 1 patient on treatment, enrollment to the trial was stopped due to discontinuation of BA development. Median age for the entire cohort was 57 years (range 51-71 years); 8 (73%) were male; and 3 of 11 (27%) were under-represented ethnic minority patients. Nine patients were ICN (Cohort 1), and 2 patients were ICR (Cohort 2). The median number of prior systemic treatments was 2 (range 1-5) across all patients, including prior ^177^Lu-DOTATATE treatment in two patients (both in Cohort 1). Baseline demographic and disease characteristics are presented in Table 1 and Supplementary Table 1; patient trial participation timepoints and status are available in Supplementary Table 2.

**Table 1:** Participant characteristics. G1: Grade 1; G2: Grade 2, G3: Grade 3; ^1^ at diagnosis; * Dural involvement status unknown

	Cohort 1 (n = 9)	Cohort 2 (n=2)	Total (n=11)
Age, years (median, range)	61 (51-71)	53.5 (52-55)	57 (51-71)
Female, n (%)	2 (22)	1 (50)	3 (27)
Race, n (%)			
White	9 (100)	2 (100)	11 (100)
Black/African American	–	–	–
Asian	–	–	–
Ethnicity, n (%)			
Not Hispanic/Latino	6 (67)	2 (100)	8 (73)
Hispanic/Latinο	3 (33)	-	3 (27)
Previous lines of therapy in metastatic setting, median (range)	2 (1–4)	4 (3–5)^a^	2 (1–5)
Kadish-INSICA stage^1^	B: 2 (22%)	B: 2 (100%)	B: 4 (36%)
	C: 4 (44%)		C: 4 (36%)
	B/C*: 2 (22%)		B/C*: 2 (18%)
	DN: 1 (11%)		DN: 1 (9%)
Hyams grade	G2: 4 (44%) G3: 4 (44%) G4: 1 (11%)	G2: 2 (100%)	G2: 6 (55%) G3: 4 (36%) G4: 1 (9%)

**Fig. 1:**
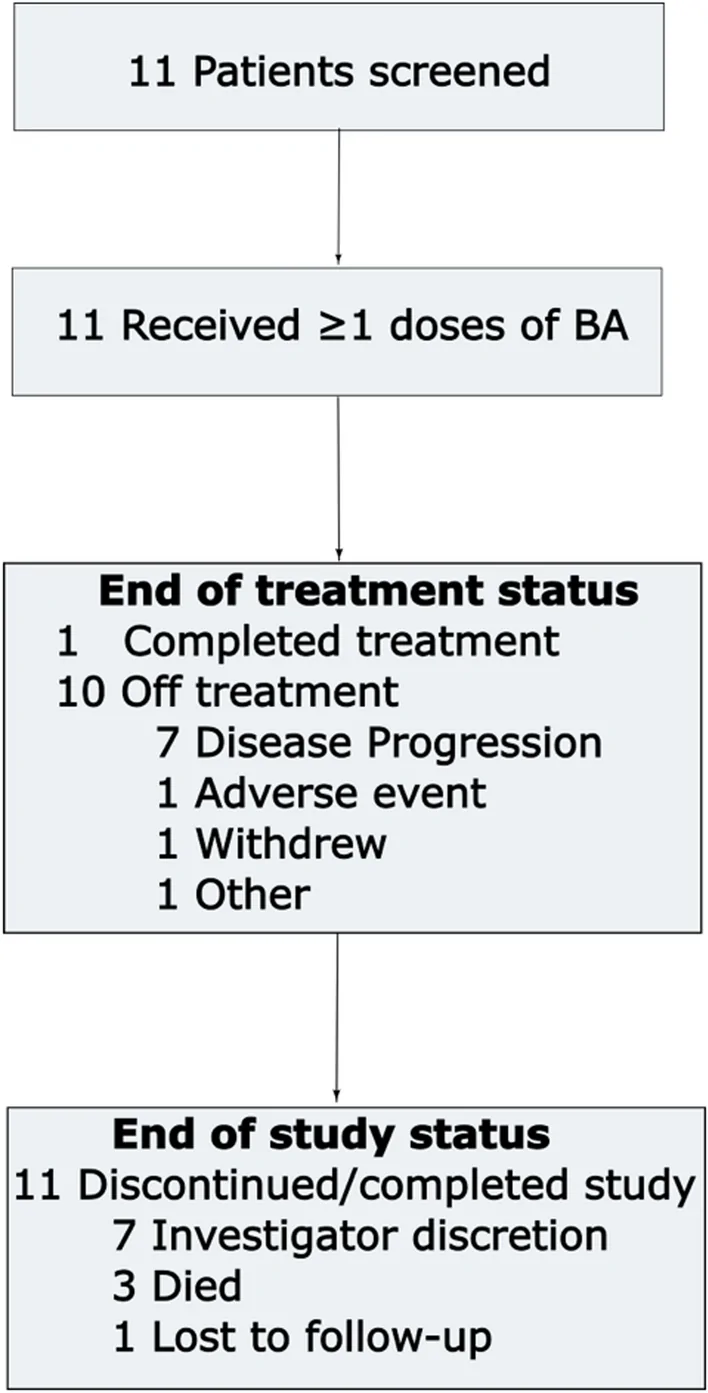
Patient flowchart

Safety was assessed in all 11 patients who received at least one dose of study treatment. Response was evaluated in 8 of 9 patients in Cohort 1 (one patient discontinued treatment shortly after receiving one dose of trial treatment due to symptomatic vasogenic brain edema (radiation-induced, preexisting) requiring incompatible medication [dexamethasone]) and 2 patients in Cohort 2. At the data cutoff date on July 15, 2024, all participants had discontinued treatment (Figure 1). The median follow-up duration for all 11 patients was 14.8 months (interquartile range (IQR): 10.5–17.5 months). In Cohort 1, the median duration of treatment (first to last treatment) was 2.8 months (IQR: 1.6–4.2 months; maximum 14.2 months). In Cohort 2, duration of treatment for the two patients was 3.9 months and 0.6 months.

### Toxicity

All 11 patients enrolled received at least one dose of treatment and were evaluable for safety. In Cohort 1 (9 patients), treatment-related adverse events (TRAEs) of any grade occurred in 8 patients (89%), and grade 3 and 4 TRAEs occurred in 2 patients (22%) (Table 2; all treatment-emergent AEs of any grade, regardless of attribution, can be found in Supplementary Table 3**)**. Most common TRAEs occurring in over 20% of patients included anemia, epistaxis, pruritus, keratoacanthoma, maculopapular rash, bruising, constipation, hypothyroidism, oral hemorrhage, and immune-mediated diabetes (Table 2; Supplementary Table 2). Grade 3 and 4 TRAEs observed were immune-mediated diabetes (2 patients [18%], one patient with preexisting type 2 diabetes) and esophageal hemorrhage (1 patient [9%], in the context of the immune-mediated diabetes presenting with diabetic ketoacidosis) (Table 2; Supplementary Table 2**)**. Serious TRAEs occurred in 2 patients (22%): immune-mediated diabetes and esophageal hemorrhage (1 patient), immune-mediated diabetes (1 patient). TRAEs led to dose reduction of BA to 300 mg in one patient (epistaxis) and to treatment discontinuation per protocol in another patient (grade 4 immune-mediated diabetes and grade 3 esophageal hemorrhage). In Cohort 2 (2 patients), 1 patient had treatment-related AEs: grade 3 anemia and epistaxis, and grade 2 diarrhea, increased lipase.

**Table 2:** Treatment-related adverse events

	Cohort 1 Patients, n (%) (N = 9)		Cohort 2 Patients, n (%) (N = 2)	
Event	Any grade^a^	Grade 3-4^b^	Any grade^c^	Grade 3-4^b^
Anemia	5 (56)	0	1 (50)	1
Epistaxis	4 (44)	0	1 (50)	1
Pruritus	4 (44)	0	0	0
Keratoacanthoma	3 (33)	0	0	0
Rash maculopapular	3 (33)	0	0	0
Bruising	2 (22)	0	0	0
Constipation	2 (22)	0	0	0
Hypothyroidism	2 (22)	0	0	0
Immune-mediated diabetes	2 (22)	2 (22)	0	0
Oral hemorrhage	2 (22)	0	0	0
Esophageal hemorrhage	1 (11)	1 (11)	0	0
Diarrhea	1 (11)	0	1 (50)	0
Lipase increase	0	0	1 (50)	0

^a^Events of any grade that occurred in ≥20% of patients; ^b^ All grade 3 and 4 events; and ^c^ Events of any grade in both patients

Across all 11 patients (both cohorts), adverse event of special interest included mucosal bleeding and cutaneous lesions possibly due to TGF-beta inhibition: epistaxis (5 patients [45%]), oral hemorrhage (3 patients [27%]), hemorrhoidal hemorrhage, and esophageal hemorrhage (1 patient [9%] each); keratoacanthoma (3 patients [27%]), maculopapular rash (1 patient [9%]). There were no treatment-related deaths.

### Clinical activity

The primary endpoint of ORR in ICN ONB patients was assessed for 8 patients in Cohort 1. There was no objective response observed by RECIST 1.1 (Table 3), but 4 patients (50%) had stable disease (SD), confirmed in 3 patients, with duration of 16.3, 12.6, and 11.5 months, without PD by the end of study follow-up in two patients. The patient with unconfirmed SD at first imaging had treatment discontinued per protocol due TRAEs but no progression reported or new treatment initiated 5.2 months following the first assessment, after which the patient was lost to follow-up.

**Table 3:** Tumor response in evaluable patients.

Response^a^	Cohort 1 (*n* = 8) *n* (%)	Cohort 2 (*n* = 2) *n* (%)
Objective response rate	0	0
Complete response	0	0
Partial response	0	0
Stable disease	4 (50.0)^b^	1 (50.0)
Progression of disease	4 (50.0)	1 (50.0)^c^

^a^Assessed by study radiologist according to the RECIST 1.1

^b^One unconfirmed SD

^c^Clinical progression

**Fig. 2:**
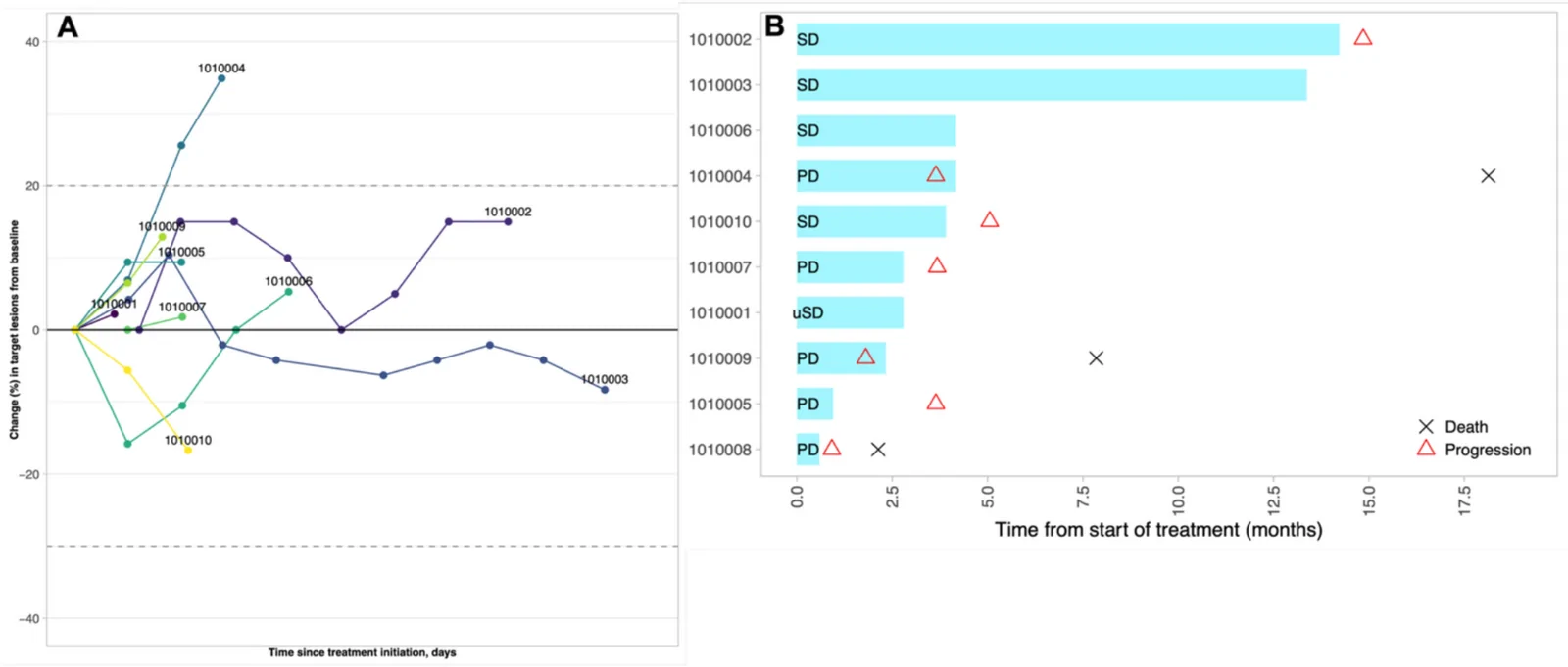
Tumor response. **A** Spider plot demonstrating changes in target lesions. **B** Swimmer plot demonstrating duration of treatment for individual patients. Cohort 2 participants: 1010008, 1010010. SD, stable disease; PD, progressive disease; and uSD, unconfirmed stable disease

Two patients were treated beyond progression per protocol, without delayed response. In Cohort 2 (ICR), one patient had confirmed SD, lasting 3.3 months. Notably, this patient’s prior ICB treatment (pembrolizumab) was discontinued not for disease progression but because of the incidental finding of lymphangioleiomyomatosis (LAM) on CT scans of the chest; there was no change in pulmonary imaging or emergence of pulmonary complaints while on treatment with BA. Spider plot demonstrating changes in target lesions and swimmer’s plot demonstrating the duration of treatment and best overall response for evaluable individual patients are shown in Fig. 2; data are available in Supplementary Table 3 and Supplementary Table 4.

**Fig. 3:**
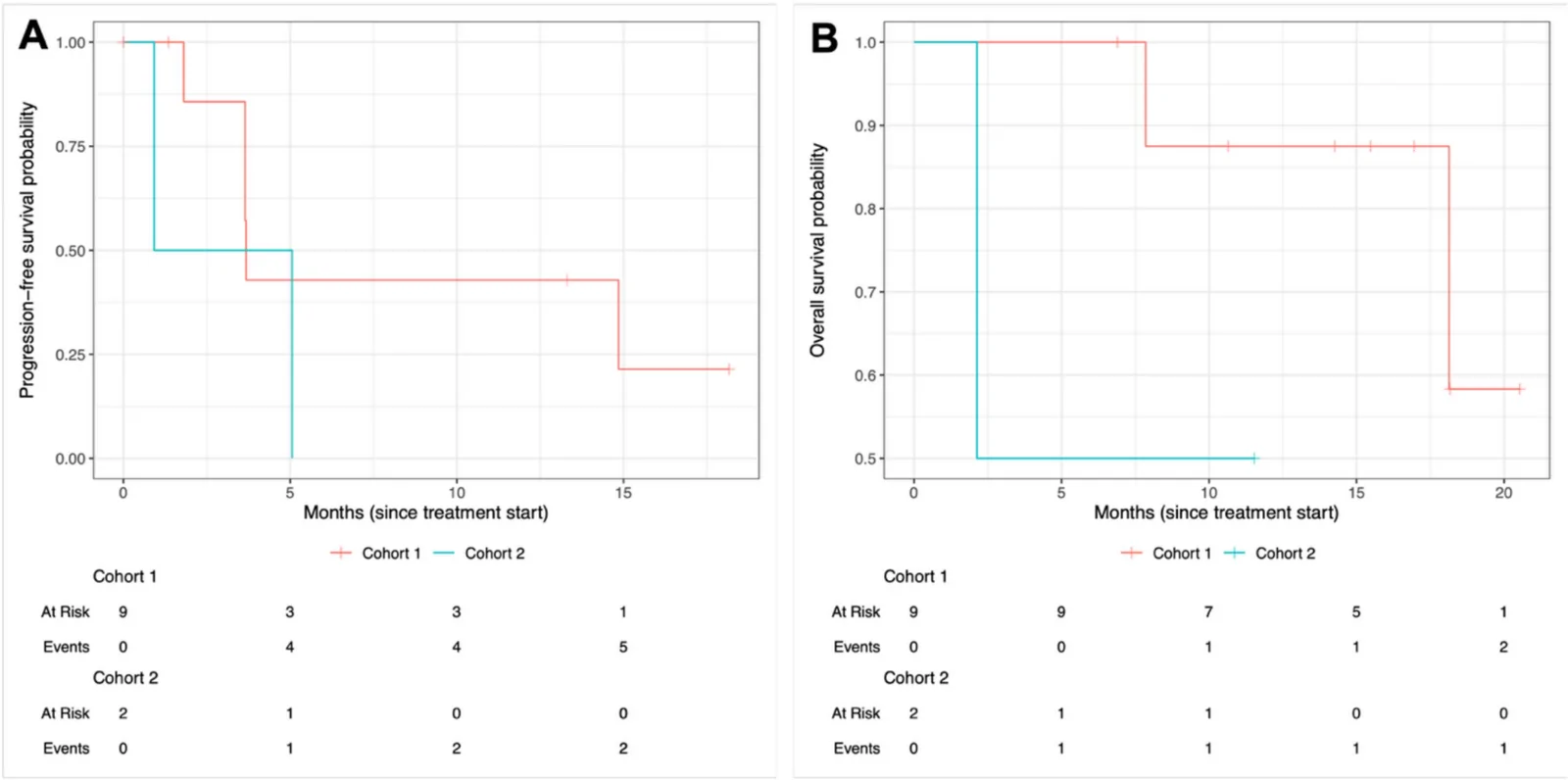
Patient survival, per cohort. **A**. Progression-free survival (PFS). **B**. Overall survival (OS). Marks on the curve indicate patients who were censored

At the end of study follow-up period, in Cohort 1 the estimated median progression-free survival (mPFS) was 3.7 months (95% CI: 1.8 months—not reached) and the estimated median OS (mOS) was not reached (95% CI: 7.9 months—not reached). In Cohort 2, the estimated mOS was 6.8 months (2.1 months to not reached) and the mPFS was 3.0 months (0.92 months to not reached). PFS and OS Kaplan–Meier (KM) curves for each cohort are shown in Fig. 3.

**Fig. 4:**
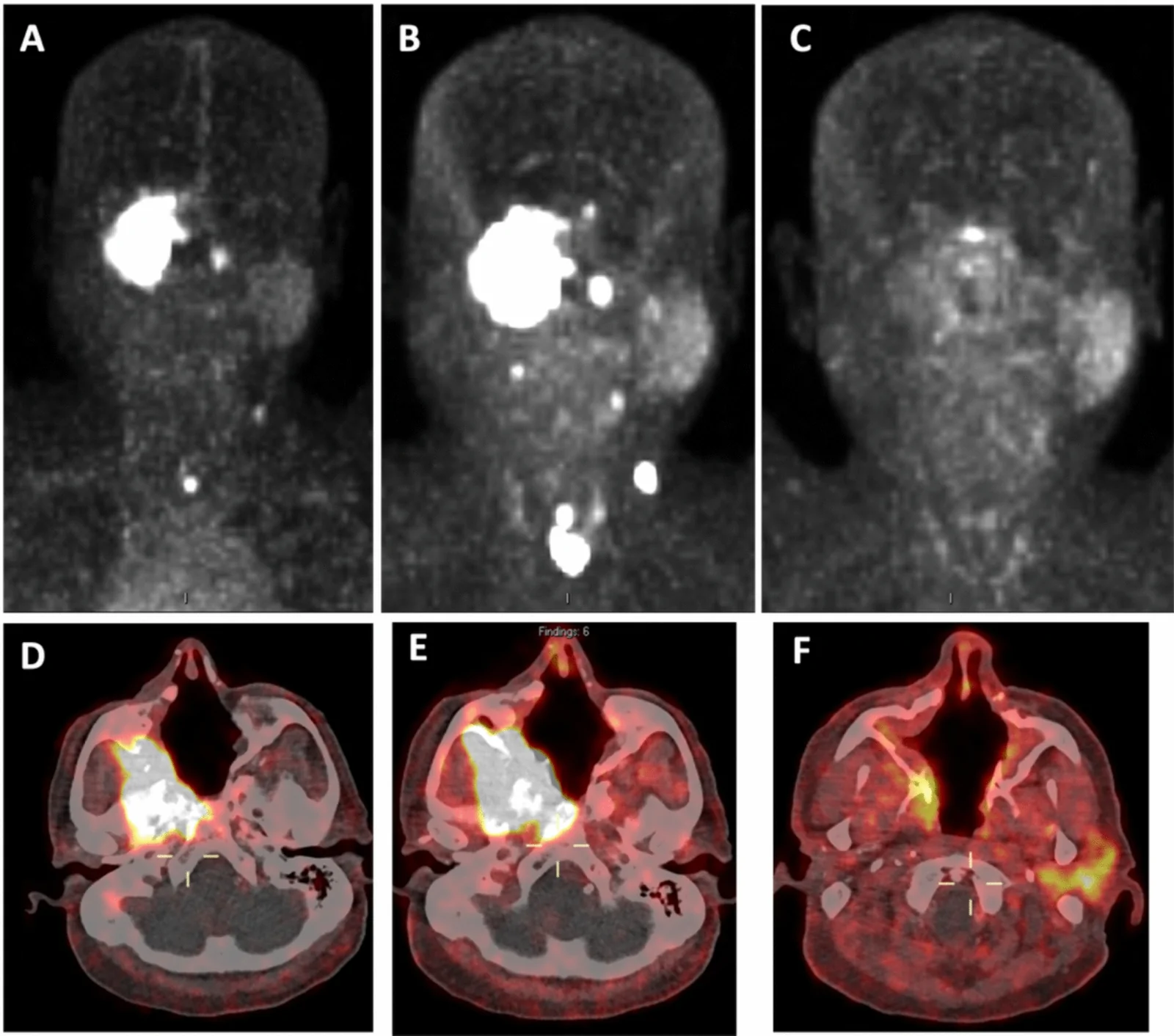
Sequential ^68^Ga-DOTATATE PET/CT imaging of patient with prolonged SD by RECIST 1.1 and metabolic response. **A-C**: Coronal PET images; **D-F**: Axial PET/CT images through plane of local recurrence. **A,D**: baseline imaging showing uptake in local recurrence and cervical lymph nodes; **B,E**: first restaging at week 9 of treatment showing increased uptake in both primary and cervical disease sites; and **C, F**: subsequent restaging at week 26 of treatment showing resolution of uptake

Notably, one patient had confirmed prolonged SD (16.3 months, ongoing at the completion of study follow-up) by RECIST 1.1 based on assessment of a target lesion in the right pterygoid fossa, which was radiotracer-avid (36.98 SUV) on baseline ^68^Ga-DOTATATE PET/CT. At the first imaging assessment (2 months after treatment start), the target lesion displayed growth of 4.2% of baseline longest diameter (on MRI). Increased radiotracer avidity to 99.03 SUV was noted on concurrent ^68^Ga-DOTATATE PET/CT; non-target small lesions also displayed increased avidity, and a few new small avid lesions were reported, considered equivocal for progression, and treatment was continued. Subsequently, the target lesion regressed gradually to -8.3% of the baseline longest dimension by the end of study follow-up. However, a biopsy of the target lesion on treatment revealed only fibrous tissue with chronic inflammation without tumor cells present, and a ^68^Ga-DOTATATE PET/CT (6 months from treatment start) revealed resolution of the tracer avid lesions (Fig. 4). Notably, this patient presented at baseline with significant trismus impeding mouth opening and oral intake since his prior radiotherapy; he reported progressively decreasing trismus and ongoing improvement in mouth opening was noted, ongoing at the end of treatment.

### Laboratory markers

Measurements of serum chromogranin A (CgA) and neuron-specific enolase (NSE) were obtained at baseline and longitudinally (Supplementary Figure 1, Supplementary Table 5). At baseline, values for CgA were available in 8 of the 11 patients and were elevated in 6 (75%) patients (reference range <93ng/ml; median 102.0 ng/mL, IQR 90.2-207.5). NSE values were available in 7 patients and were elevated in 3 (42.8%) patients (reference range ≤15 ng/mL; median 12.0 ng/mL, IQR 11.0-27.0); all 3 patients with high baseline NSE had also high baseline CgA and had PD as BOR. In the patients with available longitudinal CgA and NSE values, persistent increases in both markers, but especially in NSE accompanied tumor progression.

## Discussion

This is a single-arm phase 2 trial evaluating the efficacy of immune checkpoint blockade with simultaneous TGF-β inhibition using Bintrafusp alpha, a bifunctional TGF-β “trap”/anti-PD-L1 fusion protein, in patients with R/M ONB and showed that BA was well tolerated, with evidence of clinical benefit including a metabolic response with prolonged duration. In addition, this is the first clinical trial performed for this rare tumor, pointing to the feasibility of trials in this setting.

Given the lack of evidence on the efficacy of checkpoint blockade for patients with ONB when designing this trial, we chose a modest response rate by RECIST 1.1. of 15% as goal. Therefore, the trial used a Simon optimal two-stage to enroll a main cohort of immune checkpoint-naïve patients (Cohort 1) in two stages: first stage with 12 patients, and if one response was observed, accrual would continue to a total of 21 patients. Because of anecdotal reports of patients with R/M ONB receiving ICBs, we also included a smaller, exploratory cohort of checkpoint-resistant patients (Cohort 2, up to 8 patients), enrollment to which would stop when the needed number of participants for Cohort 1 had been accrued. While enrollment to the first stage of Cohort 1 was ongoing, development of BA was stopped, leading to interruption and then discontinuation of further enrollment to the trial by the investigator.

Consequently, both cohorts of the trial were underenrolled: Cohort 1 enrolled 9 patients (8 evaluable per RECIST 1.1), and Cohort 2 enrolled 2 patients. There was no response by RECIST 1.1. In either group therefore the trial did not meet its primary endpoint. However, in Cohort 1 there were 4 patients (50%) with SD, lasting more than 6 months in 3 of them, and almost as long in the fourth SD patient (unconfirmed, who was lost to follow-up). Notably, in one of the patients with SD as best response by RECIST 1.1, who was also one of the patients with prior ^177^Lu-DOTATATE, there was a complete and durable metabolic response on ^68^Ga-DOTATATE PET/CT, in conjunction with a biopsy from the target lesion that revealed only fibrous tissue. This indicated that the residual target lesion measured on cross-sectional imaging per RECIST 1.1 was perhaps the fibrotic compartment of the previously present tumor lesion in this patient with multiple prior surgeries and radiation therapy with extensive local fibrosis. The protocol did not prespecify criteria for PET/CT and biopsy consideration in response assessment, but these findings point to a response (which would be 1 of 8, 12.5%). In addition, this patient had significant improvement of radiotherapy-related trismus and mouth opening, potentially pointing to antifibrotic effects of TGF-β signaling blockade[32]. The estimated mPFS was 3.7 months (95% CI: 1.8 months—not reached), and the estimated mOS was not reached (95% CI: 7.9 months—not reached). In the exploratory checkpoint-resistant Cohort 2, enrollment was low, with 2 patients enrolled, one of which had SD as best response, lasting 3.3 months; this patient had an imaging finding of LAM, and treatment with BA did not cause changes in pulmonary imaging or pulmonary complaints. The estimated mOS was 6.8 months (2.1 months to not reached), and the mPFS was 3.0 months (0.92 months to not reached).

The treatment had an acceptable safety profile, with grade 3 and 4 TRAEs occurring in 2 of 9 (22%) patients in Cohort 1 (immune-mediated diabetes and esophageal hemorrhage in 1 patient, immune-mediated diabetes in another patient) and in 1 of the 2 patients in Cohort 2 (anemia, epistaxis), one TRAE-related treatment discontinuation, one dose reduction and no treatment-related deaths. TRAEs occurring in over 20% of patients included anemia, epistaxis, pruritus, keratoacanthoma, maculopapular rash, bruising, constipation, hypothyroidism, oral hemorrhage, and immune-mediated diabetes, consistent with the spectrum of known adverse events profile of BA [21, 23]. The observed incidence of immune-mediated diabetes in the trial (2 of 11 patients, 18%) is higher than the overall incidence reported in the literature for ICBs (0.45%–0.52%) [33, 34] and for BA (1.7%) [21], which should be interpreted with caution given the small sample size. Our results also show that serum CgA and NSE levels are inconsistently elevated in patients with R/M ONB, and that persistent increases, especially in serum NSE, may suggest disease progression but should be interpreted with caution and not dictate therapeutic decisions.

After the trial stopped enrolling, a retrospective case series was published of 11 R/M ONB patients who received systemic treatments between 2002 and 2022 in Japan [35]. Six patients received anti-PD-1 ICB (pembrolizumab, nivolumab); 2 responded (ORR 33.3%; 1 CR, 16.7%; 1 PR, 16.7%, both with nivolumab), and 2 patients (33.3%) with SD (both with pembrolizumab). Both responders had PD-L1 expression tumor positive score of 0. Reported immune-related AEs included typical ICB AEs: pruritus, liver function test elevation, fatigue, anorexia, diarrhea, rash, adrenal insufficiency, and hypothyroidism. The incidence of adrenal insufficiency and hypothyroidism in this cohort was 33.3% (2 patients each), higher than what is reported in the literature for PD-1 inhibitors (0.7% and 7%) [36] but due to the small sample size this should be interpreted with caution.

Our results align with the published reports about ICBs in R/M ONB, indicating limited activity [20, 35] and support a role for such treatment in the appropriate context, especially considering the limited therapeutic options. In a research setting, combinations of ICBs with other agents are likely required for increased activity. Considering the lack of ONB preclinical models, rational combinations of immunotherapy may be informed by ONB translational research findings, such as augmenting the tumor cell MHC class II, CXCL9, or CXCL10 expression [37] or inhibiting EZH2 activity [38], or by clinical observations, e.g., with targeted radioligands such as 177Lu-DOTATATE, a prior treatment in the patient with complete metabolic response in this trial but also active as monotherapy in ONB. Research on systemic treatments for R/M ONB may also consider agents successful in related tumors, e.g., agents targeting delta-like ligand 3 (DLL3) or other surface molecules in transcriptomically similar cancers, e.g., small cell lung cancer [39, 40] or in other tumors of neuroectodermal origin, such as anti-GD2-monoclonal antibodies in neuroblastoma [41].

A limitation of our study is the small sample size due to the incomplete enrollment, which imposes limitations to the interpretation of the data. Exploratory analyses of research blood samples and tumor biospecimens are ongoing to characterize the changes induced by the treatment.

Notably, despite the rarity of ONB, all 11 patients were enrolled in 1 year, showing the feasibility of a clinical trial addressing a rare tumor. Further, 3 of 11 (27%) patients were from under-represented ethnic minority (Hispanic or Latino), surpassing the reported 7.1% rate for NCI protocol participants (2005–2020) [42] or 7% reported for trials of chordoma, another rare tumor [43]. The fast accrual and increased representation of ethnic minority patients may be reflecting effects of the mechanism of this trial (single-site trial conducted by the intramural NCI at the NIH Clinical Center in Bethesda, Maryland, without cost to participants or billing of third-party payers, and assistance provided for travel expenses of participants) and of community outreach efforts such as the Olfactory Neuroblastoma Community Conference Series and Patient Day event, organized annually (since 2020) by the NCI Sinonasal and Skull Base Tumor Program [44].

In conclusion, concurrent blockade of PD-L1 and the TGF-β with BA had a manageable safety profile and resulted in prolonged disease control with evidence of clinical activity in patients with R/M ONB, supporting further exploration of combinatorial immunotherapy approaches in ONB. Our results also show the feasibility of interventional clinical trials for ONB, an unmet need.

## Supplementary Information

Below is the link to the electronic supplementary material.Supplementary file1 (DOCX 395 KB)

## Data Availability

Deidentified participant level data are available with the Supplementary Material.

## Abbreviations

BA: Bintrafusp alfa
CgA: Chromogranin A
CR: Complete response
CT: Computed tomography
CTCAE: Common terminology criteria for adverse events
DOR: Duration of response
DLL3: Delta-like ligand 3
ECOG: Eastern cooperative oncology group
EZH2: Enhancer of zeste homolog 2
HPV: Human Papilloma Virus
ICB: Immune checkpoint blockade
ICN: ICB-naïve
ICR: ICB-resistant
IQR: Interquartile range
IRB: Institutional review board
KM: Kaplan–Meier
LAM: Lymphangioleiomyomatosis
MHC: Major histocompatibility complex
mOS: Median overall survival
mPFS: Median progression-free survival
MRI: Magnetic resonance imaging
NCI: National cancer institute
NE: Neuroendocrine
NIH: National Institutes of Health
NSE: Neuron-specific enolase
ONB: Olfactory neuroblastoma
ORR: Overall response rate
OS: Overall survival
PD-L1: Programmed death ligand 1
PET/CT: Positron emission tomography/computed tomography
PFS: Progression-free survival
PR: Partial response
PS: Performance status
R/M: Recurrent or metastatic
RECIST: Response evaluation criteria in solid tumors
SD: Stable disease
SSTR2: Somatostatin receptor type 2
TGF-β: Transforming growth factor beta
TRAEs: Treatment-related adverse events
uSD: Unconfirmed SD
