# Supplementary material for: Bintrafusp alfa for patients with recurrent or metastatic olfactory neuroblastoma
Source: Cancer Immunol Immunother. 2026 Jun 17;75(9):218. doi: 10.1007/s00262-026-04462-4 (PMC13522318; doi:10.1007/s00262-026-04462-4)
Supplement: Supplementary file 1 [file 262_2026_4462_MOESM1_ESM.docx]

**Cancer Immunology, Immunotherapy (submitted in 2026) – Nyall London et al.**

**SUPPLEMENTARY FIGURES**

**Supplementary Figure 1. Longitudinal serum chromogranin A (CgA) and neuron-specific enolase (NSE) per patient.** A. Serum CgA. B. Serum NSE. In both panels the horizontal dotted line represents the upper limit of normal for the assay. Green: the plot for the patient labeled. Grey: plots for the other patients.

**SUPPLEMENTARY TABLES**

| **Patient** | **Cohort** | **BOR** | **KM stage** | **KI stage** | **Hyams grade** | **Disease sites** |
| --- | --- | --- | --- | --- | --- | --- |
| 1010001 | 1 | uSD | C | C | 3 | Primary site, Meningeal spread, Regional lymph nodes |
| 1010002 | 1 | SD | C | C | 3 | Primary site, Regional soft tissue |
| 1010003 | 1 | SD* | D | DN | 3 | Primary site, Regional lymph nodes |
| 1010004 | 1 | PD | C | C | 2 | Primary site, Regional lymph nodes, Meningeal spread, Ventricular system |
| 1010005 | 1 | PD | C | C | 2 | Primary site, Meningeal spread, Drop lesions, Thyroid gland |
| 1010006 | 1 | SD | C | B/C^†^ | 2 | Primary site, Regional lymph nodes, Regional soft tissue, Meningeal spread, Osseous lesions |
| 1010007 | 1 | PD | C | B/C^†^ | 2 | Primary site, Regional soft tissue, Osseous lesions |
| 1010008 | 2 | PD | C | B | 2 | Primary site, Regional lymph nodes, Distant lymph nodes, Pulmonary lesions, Distant soft tissue lesions, Osseous lesions |
| 1010009 | 1 | PD | C | B | 3 | Osseous lesions, Distant lymph nodes |
| 1010010 | 2 | SD | C | B | 2 | Primary site, Regional lymph nodes, Meningeal spread, Drop lesions |
| 1010011 | 1 | NE | C | B | 4 | Primary site, Meningeal spread, Osseous lesions |

**Supplementary Table 1.** Patient characteristics. BOR: best overall response. KM stage: Kadish-Morita stage; KI stage: Kadish-INSICA stage. ^†^Dural involvement status unknown. *Patient with SD by RECIST 1.1, complete metabolic response, biopsy of target lesion with fibrosis, no residual tumor. Patients 1010003 and 1010009 had received prior ^177^Lu-DOTATATE treatment.

| **Cohort** | **Patient** | **First treatment** | **Last treatment** | **Starting dose** | **Dose reduction** | **Off treatment date** | **Off treatment reason** | **Off study** | **Off study reason** | **BOR** | **BOR date** | **Progression** | **Progression date** | **Death** | **Date of death** | **Last follow-up** |
| --- | --- | --- | --- | --- | --- | --- | --- | --- | --- | --- | --- | --- | --- | --- | --- | --- |
| 1 | 1010001 | 2022-06-21 | 2022-09-13 | 1200mg | no | 2022-11-16 | Adverse Event | 2023-05-04 | Lost To Follow-up | uSD | 2022-08-01 | no |  | no |  | 2023-01-17 |
| 1 | 1010002 | 2022-09-22 | 2023-11-28 | 1200mg | no | 2023-12-19 | Disease Progression | 2024-05-23 | PI discretion | SD | 2022-11-28 | yes | 2023-12-18 | no |  | 2024-06-08 |
| 1 | 1010003 | 2022-10-11 | 2023-11-21 | 1200mg | no | 2024-02-13 | Completion of protocol therapy | 2024-07-09 | PI discretion | SD | 2022-12-06 | no |  | no |  | 2024-04-16 |
| 1 | 1010004 | 2022-10-25 | 2023-02-28 | 1200mg | no | 2023-03-30 | Disease Progression | 2024-05-02 | Death | PD | 2023-02-13 | yes | 2023-02-13 | yes | 2024-04-29 | 2024-04-29 |
| 1 | 1010005 | 2022-12-06 | 2023-01-03 | 1200mg | no | 2023-03-28 | Disease Progression | 2024-05-02 | PI discretion | PD | 2023-03-27 | yes | 2023-03-27 | no |  | 2024-05-05 |
| 1 | 1010006 | 2023-02-07 | 2023-06-13 | 1200mg | yes | 2023-09-19 | Patient preference | 2024-05-23 | PI discretion | SD | 2023-04-03 | no |  | no |  | 2024-05-23 |
| 1 | 1010007 | 2023-02-07 | 2023-05-02 | 1200mg | no | 2023-05-30 | Disease Progression | 2024-05-02 | PI discretion | PD | 2023-05-30 | yes | 2023-05-30 | no |  | 2024-04-16 |
| 2 | 1010008 | 2023-02-28 | 2023-03-17 | 1200mg | no | 2023-03-28 | Disease Progression | 2023-05-04 | Death | PD | 2023-03-28 | yes | 2023-03-28 | yes | 2023-05-04 | 2023-05-04 |
| 1 | 1010009 | 2023-05-09 | 2023-07-18 | 1200mg | no | 2023-08-08 | Disease Progression | 2024-01-03 | Death | PD | 2023-07-03 | yes | 2023-07-03 | yes | 2024-01-03 | 2024-01-03 |
| 2 | 1010010 | 2023-05-17 | 2023-09-12 | 1200mg | no | 2023-10-18 | Disease Progression | 2024-05-02 | PI discretion | SD | 2023-07-11 | yes | 2023-10-18 | no |  | 2024-05-02 |
| 1 | 1010011 | 2023-06-13 | 2023-06-13 | 1200mg | no | 2023-08-31 | Intercurrent illness-incompatible medication | 2024-05-02 | PI discretion | NE |  | no |  | no | 2025-12-09 | 2024-05-02 |

**Supplementary Table 2.** Patient trial timepoints and status. BOR: best overall response.

| **Patient ID** | **Cohort** | **CTCAE** | **SOC** | **AE Description** | **AE Description** | **Grade** | **Attr to Bintrafusp alfa** | **Attr to Disease** | **Attr to Other** | **Other, Specify** | **AESI** | **Serious Y/N** | **Action** |
| --- | --- | --- | --- | --- | --- | --- | --- | --- | --- | --- | --- | --- | --- |
| 1010001 | 1/Cohort 1 | Atrioventricular block first degree | Cardiac disorders |  |  | 2:Moderate Adverse Event | Adverse Event Related | Adverse Event Unrelated | Adverse Event Related | DKA Crisis; elevated troponin | No | No | Dose not changed |
| 1010001 | 1/Cohort 1 | Acute kidney injury | Renal and urinary disorders |  |  | 2:Moderate Adverse Event | Adverse Event Related | Adverse Event Unrelated | Adverse Event Related | DKA | No | No | Dose not changed |
| 1010001 | 1/Cohort 1 | Cardiac troponin I increased | Investigations |  |  | 1:Mild Adverse Event | Adverse Event Related | Adverse Event Unrelated | Adverse Event Related | DKA | No | No | Dose not changed |
| 1010001 | 1/Cohort 1 | Electrocardiogram T wave abnormal | Investigations |  |  | 2:Moderate Adverse Event | Adverse Event Related | Adverse Event Unrelated | Adverse Event Related | Atrioventricular block 1st degree | No | No | Dose not changed |
| 1010001 | 1/Cohort 1 | Esophageal hemorrhage | Gastrointestinal disorders |  |  | 3:Severe Adverse Event | Adverse Event Related | Adverse Event Unrelated | Adverse Event Related | Shock | Yes | Yes | Dose not changed |
| 1010001 | 1/Cohort 1 | Metabolism and nutrition disorders | Metabolism and nutrition disorders | Immune-mediated diabetes | Immune-mediated diabetes | 4:Life Threatening or Disabling Adverse Event | Adverse Event Related | Adverse Event Unrelated | Adverse Event Related | HX- DM; DKA: acidosis & hypovolemia | No | Yes | Not Applicable |
| 1010001 | 1/Cohort 1 | Diarrhea | Gastrointestinal disorders |  |  | 1:Mild Adverse Event | Adverse Event Related | Adverse Event Unrelated | |  | No | No | Dose not changed |
| 1010002 | 1/Cohort 1 | Pruritus | Skin and subcutaneous tissue disorders |  |  | 2:Moderate Adverse Event | Adverse Event Related | Adverse Event Unrelated | |  | No | No | Dose not changed |
| 1010002 | 1/Cohort 1 | Pleural effusion | Respiratory, thoracic and mediastinal disorders |  |  | 2:Moderate Adverse Event | Adverse Event Unrelated | Adverse Event Related | |  | No | No | Dose interrupted |
| 1010002 | 1/Cohort 1 | Neoplasms benign, malignant and unspecified | Neoplasms benign, malignant and unspecified | Keratoacanthoma; Located on left shin, left ear | Keratoacanthoma | 1:Mild Adverse Event | Adverse Event Related | Adverse Event Unrelated | |  | Yes | No | Dose not changed |
| 1010002 | 1/Cohort 1 | Respiratory, thoracic and mediastinal disorders | Respiratory, thoracic and mediastinal disorders | Pneumomediastinum, seen on CT 1/9/23 | Pneumomediastinum | 1:Mild Adverse Event | Adverse Event Unrelated | Adverse Event Related | |  | No | No | Dose not changed |
| 1010002 | 1/Cohort 1 | Insomnia | Psychiatric disorders | Due to itching/skin changes caused by M7824 |  | 1:Mild Adverse Event | Adverse Event Related | Adverse Event Unrelated | |  | No | No | Dose not changed |
| 1010002 | 1/Cohort 1 | Rash maculo-papular | Skin and subcutaneous tissue disorders |  |  | 1:Mild Adverse Event | Adverse Event Related | Adverse Event Unrelated | |  | Yes | No | Dose not changed |
| 1010002 | 1/Cohort 1 | Dyspepsia | Gastrointestinal disorders |  |  | 1:Mild Adverse Event | Adverse Event Unrelated | Adverse Event Unrelated | Adverse Event Related | Pantoprazole | No | No | Dose not changed |
| 1010002 | 1/Cohort 1 | Pneumonitis | Respiratory, thoracic and mediastinal disorders | Seen on chest CT 2/7/23 |  | 1:Mild Adverse Event | Adverse Event Related | Adverse Event Unrelated | |  | No | No | Dose not changed |
| 1010002 | 1/Cohort 1 | Oral hemorrhage | Gastrointestinal disorders | Intermittent bleeding from gums |  | 1:Mild Adverse Event | Adverse Event Related | Adverse Event Unrelated | |  | Yes | No | Dose not changed |
| 1010002 | 1/Cohort 1 | Sinus tachycardia | Cardiac disorders |  |  | 1:Mild Adverse Event | Adverse Event Unrelated | Adverse Event Unrelated | Adverse Event Related | Stress; Thyroid treatment | No | No | Dose not changed |
| 1010002 | 1/Cohort 1 | Pruritus | Skin and subcutaneous tissue disorders | Rash on torso |  | 2:Moderate Adverse Event | Adverse Event Related | Adverse Event Unrelated | |  | No | No | Dose not changed |
| 1010002 | 1/Cohort 1 | Pruritus | Skin and subcutaneous tissue disorders |  |  | 1:Mild Adverse Event | Adverse Event Related | Adverse Event Unrelated | |  | No | No | Dose not changed |
| 1010002 | 1/Cohort 1 | Pneumonitis | Respiratory, thoracic and mediastinal disorders |  |  | 1:Mild Adverse Event | Adverse Event Related | Adverse Event Unrelated | |  | No | No | Dose interrupted |
| 1010002 | 1/Cohort 1 | Pneumonitis | Respiratory, thoracic and mediastinal disorders |  |  | 2:Moderate Adverse Event | Adverse Event Related | Adverse Event Unrelated | |  | No | No | Dose interrupted |
| 1010002 | 1/Cohort 1 | Hypertension | Vascular disorders |  |  | 1:Mild Adverse Event | Adverse Event Unrelated | Adverse Event Unrelated | Adverse Event Related | Age | No | No | Dose not changed |
| 1010002 | 1/Cohort 1 | Dry eye | Eye disorders |  |  | 2:Moderate Adverse Event | Adverse Event Related | Adverse Event Unrelated | |  | No | No | Dose not changed |
| 1010002 | 1/Cohort 1 | Epistaxis | Respiratory, thoracic and mediastinal disorders |  |  | 2:Moderate Adverse Event | Adverse Event Related | Adverse Event Related | |  | Yes | No | Dose not changed |
| 1010002 | 1/Cohort 1 | Gastrointestinal disorders | Gastrointestinal disorders | Diverticulitis | Diverticulitis | 2:Moderate Adverse Event | Adverse Event Unrelated | Adverse Event Unrelated | Adverse Event Related | h/o diverticulosis | No | No | Dose interrupted |
| 1010002 | 1/Cohort 1 | Eye infection | Infections and infestations |  |  | 2:Moderate Adverse Event | Adverse Event Unrelated | Adverse Event Unrelated | Adverse Event Related | Dry eye | No | No | Dose not changed |
| 1010002 | 1/Cohort 1 | Bruising | Injury, poisoning and procedural complications | Left forearm |  | 1:Mild Adverse Event | Adverse Event Related | Adverse Event Related | |  | No | No | Dose not changed |
| 1010002 | 1/Cohort 1 | Folliculitis | Infections and infestations | Rash on torso and back |  | 2:Moderate Adverse Event | Adverse Event Related | Adverse Event Unrelated | |  | No | No | Dose not changed |
| 1010002 | 1/Cohort 1 | Hypothyroidism | Endocrine disorders |  |  | 2:Moderate Adverse Event | Adverse Event Related | Adverse Event Related | |  | No | No | Dose not changed |
| 1010002 | 1/Cohort 1 | Folliculitis | Infections and infestations | Left shoulder; neck |  | 1:Mild Adverse Event | Adverse Event Related | Adverse Event Unrelated | |  | No | No | Dose not changed |
| 1010003 | 1/Cohort 1 | Neoplasms benign, malignant and unspecified | Neoplasms benign, malignant and unspecified | Keratocanthoma; Forearms and upper back | Keratoacanthoma | 1:Mild Adverse Event | Adverse Event Related | Adverse Event Unrelated | |  | Yes | No | Dose not changed |
| 1010003 | 1/Cohort 1 | Rash maculo-papular | Skin and subcutaneous tissue disorders |  |  | 1:Mild Adverse Event | Adverse Event Related | Adverse Event Unrelated | Adverse Event Related | Insect bites; Itchy rash-torso,legs,arms | No | No | Dose not changed |
| 1010003 | 1/Cohort 1 | Epistaxis | Respiratory, thoracic and mediastinal disorders | Considered non-SAE AESI per protocol |  | 2:Moderate Adverse Event | Adverse Event Related | Adverse Event Related | |  | Yes | No | Dose not changed |
| 1010003 | 1/Cohort 1 | Rash maculo-papular | Skin and subcutaneous tissue disorders |  |  | 1:Mild Adverse Event | Adverse Event Unrelated | Adverse Event Unrelated | Adverse Event Related | Insect bites | No | No | Dose not changed |
| 1010003 | 1/Cohort 1 | Headache | Nervous system disorders | Intermittent, 3-4x weekly |  | 2:Moderate Adverse Event | Adverse Event Related | Adverse Event Related | |  | No | No | Dose not changed |
| 1010003 | 1/Cohort 1 | Creatinine increased | Investigations |  |  | 2:Moderate Adverse Event | Adverse Event Unrelated | Adverse Event Unrelated | Adverse Event Related | Diabetes | No | No | Dose not changed |
| 1010003 | 1/Cohort 1 | Epistaxis | Respiratory, thoracic and mediastinal disorders | Small, intermittent; Resolved with pressure |  | 1:Mild Adverse Event | Adverse Event Related | Adverse Event Related | |  | Yes | No | Dose not changed |
| 1010003 | 1/Cohort 1 | Pharyngeal mucositis | Respiratory, thoracic and mediastinal disorders |  |  | 1:Mild Adverse Event | Adverse Event Related | Adverse Event Related | |  | No | No | Dose interrupted |
| 1010003 | 1/Cohort 1 | Neoplasms benign, malignant and unspecified | Neoplasms benign, malignant and unspecified | Keratoacanthoma | Keratoacanthoma | 2:Moderate Adverse Event | Adverse Event Related | Adverse Event Unrelated | |  | Yes | No | Dose interrupted |
| 1010003 | 1/Cohort 1 | Pruritus | Skin and subcutaneous tissue disorders |  |  | 2:Moderate Adverse Event | Adverse Event Related | Adverse Event Unrelated | |  | No | No | Dose not changed |
| 1010003 | 1/Cohort 1 | Neoplasms benign, malignant and unspecified | Neoplasms benign, malignant and unspecified | Basal cell carcinoma; Cervical lesions | Basal cell carcinoma | 2:Moderate Adverse Event | Adverse Event Unrelated | Adverse Event Unrelated | Adverse Event Related | Sun exposure | No | No | Dose not changed |
| 1010003 | 1/Cohort 1 | Arthralgia | Musculoskeletal and connective tissue disorders | Left elbow |  | 1:Mild Adverse Event | Adverse Event Unrelated | Adverse Event Unrelated | Adverse Event Related | Overuse of joint | No | No | Dose not changed |
| 1010003 | 1/Cohort 1 | Skin and subcutaneous tissue disorders | Skin and subcutaneous tissue disorders | Milia cysts | Milia cysts | 1:Mild Adverse Event | Adverse Event Unrelated | Adverse Event Unrelated | Adverse Event Related | Sun exposure | No | No | Dose not changed |
| 1010003 | 1/Cohort 1 | Anemia | Blood and lymphatic system disorders |  |  | 2:Moderate Adverse Event | Adverse Event Related | Adverse Event Unrelated | |  | No | No | Dose not changed |
| 1010003 | 1/Cohort 1 | Myalgia | Musculoskeletal and connective tissue disorders |  |  | 1:Mild Adverse Event | Adverse Event Unrelated | Adverse Event Unrelated | Adverse Event Related | Aging; Physical activity | No | No | Dose not changed |
| 1010003 | 1/Cohort 1 | Hypertension | Vascular disorders |  |  | 3:Severe Adverse Event | Adverse Event Unrelated | Adverse Event Unrelated | Adverse Event Related | Diabetes; Hx hypertension | No | No | Dose interrupted |
| 1010003 | 1/Cohort 1 | Epistaxis | Respiratory, thoracic and mediastinal disorders |  |  | 2:Moderate Adverse Event | Adverse Event Related | Adverse Event Related | |  | Yes | No | Dose interrupted |
| 1010003 | 1/Cohort 1 | Bruising | Injury, poisoning and procedural complications | Left arm |  | 1:Mild Adverse Event | Adverse Event Related | Adverse Event Unrelated | |  | No | No | Dose not changed |
| 1010003 | 1/Cohort 1 | Platelet count decreased | Investigations |  |  | 1:Mild Adverse Event | Adverse Event Related | Adverse Event Unrelated | |  | No | No | Dose not changed |
| 1010003 | 1/Cohort 1 | Allergic rhinitis | Respiratory, thoracic and mediastinal disorders |  |  | 2:Moderate Adverse Event | Adverse Event Unrelated | Adverse Event Unrelated | Adverse Event Related | seasonal allergy exposure | No | No | Dose not changed |
| 1010003 | 1/Cohort 1 | Pruritus | Skin and subcutaneous tissue disorders | Reported on scalp 4/11/23 |  | 1:Mild Adverse Event | Adverse Event Related | Adverse Event Unrelated | |  | No | No | Dose not changed |
| 1010003 | 1/Cohort 1 | Skin and subcutaneous tissue disorders | Skin and subcutaneous tissue disorders | Basal cell carcinoma; On right chest | Basal cell carcinoma | 2:Moderate Adverse Event | Adverse Event Unrelated | Adverse Event Unrelated | Adverse Event Related | Sun exposure | No | No | Dose not changed |
| 1010004 | 1/Cohort 1 | Ear pain | Ear and labyrinth disorders | Left ear fullness |  | 1:Mild Adverse Event | Adverse Event Unrelated | Adverse Event Related | |  | No | No | Dose not changed |
| 1010004 | 1/Cohort 1 | Oral dysesthesia | Gastrointestinal disorders | After receiving study infusions |  | 1:Mild Adverse Event | Adverse Event Related | Adverse Event Related | |  | No | No | Dose not changed |
| 1010004 | 1/Cohort 1 | Anemia | Blood and lymphatic system disorders |  |  | 2:Moderate Adverse Event | Adverse Event Related | Adverse Event Unrelated | Adverse Event Related | Hemorrhoids | No | No | Dose interrupted |
| 1010004 | 1/Cohort 1 | Hematuria | Renal and urinary disorders |  |  | 1:Mild Adverse Event | Adverse Event Unrelated | Adverse Event Unrelated | Adverse Event Related | Previous prostate treatment | Yes | No | Dose not changed |
| 1010004 | 1/Cohort 1 | Hemorrhoidal hemorrhage | Gastrointestinal disorders |  |  | 1:Mild Adverse Event | Adverse Event Related | Adverse Event Unrelated | Adverse Event Related | Crohn's disease | Yes | No | Dose not changed |
| 1010004 | 1/Cohort 1 | Neoplasms benign, malignant and unspecified | Neoplasms benign, malignant and unspecified | Keratoacanthoma | Keratoacanthoma | 1:Mild Adverse Event | Adverse Event Related | Adverse Event Unrelated | |  | Yes | No | Dose not changed |
| 1010004 | 1/Cohort 1 | Epistaxis | Respiratory, thoracic and mediastinal disorders | Intermittent |  | 1:Mild Adverse Event | Adverse Event Related | Adverse Event Related | |  | Yes | No | Dose not changed |
| 1010005 | 1/Cohort 1 | Anemia | Blood and lymphatic system disorders |  |  | 1:Mild Adverse Event | Adverse Event Related | Adverse Event Related | |  | No | No | Dose not changed |
| 1010005 | 1/Cohort 1 | Folliculitis | Infections and infestations | On face |  | 1:Mild Adverse Event | Adverse Event Unrelated | Adverse Event Unrelated | Adverse Event Related | Mask use | No | No | Dose not changed |
| 1010005 | 1/Cohort 1 | Eye disorders | Eye disorders | Corneal perforation | Corneal perforation | 4:Life Threatening or Disabling Adverse Event | Adverse Event Unrelated | Adverse Event Related | Adverse Event Unrelated | | No | Yes | Dose interrupted |
| 1010005 | 1/Cohort 1 | Anemia | Blood and lymphatic system disorders |  |  | 2:Moderate Adverse Event | Adverse Event Related | Adverse Event Related | Adverse Event Related | Surgical repair (eye) | No | No | Dose not changed |
| 1010005 | 1/Cohort 1 | Esophagitis | Gastrointestinal disorders |  |  | 2:Moderate Adverse Event | Adverse Event Unrelated | Adverse Event Unrelated | Adverse Event Related | Radiation treatment | No | No | Dose interrupted |
| 1010005 | 1/Cohort 1 | Weight loss | Investigations |  |  | 1:Mild Adverse Event | Adverse Event Unrelated | Adverse Event Related | Adverse Event Related | Esophagitis | No | No | Dose not changed |
| 1010005 | 1/Cohort 1 | Urinary tract infection | Infections and infestations |  |  | 2:Moderate Adverse Event | Adverse Event Unrelated | Adverse Event Unrelated | Adverse Event Related | Radiation therapy | No | No | Dose not changed |
| 1010006 | 1/Cohort 1 | Anemia | Blood and lymphatic system disorders |  |  | 1:Mild Adverse Event | Adverse Event Related | Adverse Event Related | |  | No | No | Dose reduced |
| 1010006 | 1/Cohort 1 | Lymphedema | Vascular disorders | Fullness/mild swelling to central neck |  | 1:Mild Adverse Event | Adverse Event Unrelated | Adverse Event Related | |  | No | No | Dose not changed |
| 1010006 | 1/Cohort 1 | Rash maculo-papular | Skin and subcutaneous tissue disorders |  |  | 1:Mild Adverse Event | Adverse Event Related | Adverse Event Unrelated | |  | No | No | Dose not changed |
| 1010006 | 1/Cohort 1 | Pruritus | Skin and subcutaneous tissue disorders |  |  | 1:Mild Adverse Event | Adverse Event Related | Adverse Event Unrelated | |  | No | No | Dose not changed |
| 1010006 | 1/Cohort 1 | Pruritus | Skin and subcutaneous tissue disorders |  |  | 2:Moderate Adverse Event | Adverse Event Related | Adverse Event Unrelated | |  | No | No | Dose not changed |
| 1010006 | 1/Cohort 1 | Tooth infection | Infections and infestations |  |  | 2:Moderate Adverse Event | Adverse Event Unrelated | Adverse Event Unrelated | Adverse Event Related | Dental caries | No | No | Dose not changed |
| 1010006 | 1/Cohort 1 | Hypothyroidism | Endocrine disorders |  |  | 2:Moderate Adverse Event | Adverse Event Related | Adverse Event Unrelated | |  | No | No | Dose reduced |
| 1010006 | 1/Cohort 1 | Fever | General disorders and administration site conditions |  |  | 1:Mild Adverse Event | Adverse Event Unrelated | Adverse Event Unrelated | Adverse Event Related | Viral illness | No | No | Dose not changed |
| 1010006 | 1/Cohort 1 | Skin hypopigmentation | Skin and subcutaneous tissue disorders | Vitiligo - backs of hands and right side of neck | Vitiligo | 1:Mild Adverse Event | Adverse Event Related | Adverse Event Unrelated | |  | No | No | Dose not changed |
| 1010006 | 1/Cohort 1 | Constipation | Gastrointestinal disorders |  |  | 1:Mild Adverse Event | Adverse Event Related | Adverse Event Unrelated | |  | No | No | Dose not changed |
| 1010006 | 1/Cohort 1 | Facial pain | General disorders and administration site conditions |  |  | 1:Mild Adverse Event | Adverse Event Unrelated | Adverse Event Unrelated | Adverse Event Related | Dental extraction | No | No | Dose not changed |
| 1010006 | 1/Cohort 1 | Flu like symptoms | General disorders and administration site conditions | Achiness, Weakness, Nausea |  | 1:Mild Adverse Event | Adverse Event Related | Adverse Event Related | |  | No | No | Dose not changed |
| 1010006 | 1/Cohort 1 | Metabolism and nutrition disorders | Metabolism and nutrition disorders | Immune-mediated diabetes | Immune-mediated diabetes | 3:Severe Adverse Event | Adverse Event Related | Adverse Event Unrelated | |  | No | Yes | Dose interrupted |
| 1010006 | 1/Cohort 1 | Alanine aminotransferase increased | Investigations |  |  | 1:Mild Adverse Event | Adverse Event Related | Adverse Event Unrelated | Adverse Event Related | Diabetes | No | No | Dose not changed |
| 1010006 | 1/Cohort 1 | Aspartate aminotransferase increased | Investigations |  |  | 1:Mild Adverse Event | Adverse Event Related | Adverse Event Related | Adverse Event Related | Diabetes | No | No | Dose not changed |
| 1010006 | 1/Cohort 1 | Hyponatremia | Metabolism and nutrition disorders |  |  | 2:Moderate Adverse Event | Adverse Event Unrelated | Adverse Event Unrelated | Adverse Event Related | Diabetes | No | No | Dose not changed |
| 1010006 | 1/Cohort 1 | Arthralgia | Musculoskeletal and connective tissue disorders | Left hip |  | 1:Mild Adverse Event | Adverse Event Unrelated | Adverse Event Unrelated | Adverse Event Related | Aging, Degenerative left hip | No | No | Dose not changed |
| 1010006 | 1/Cohort 1 | Pain | General disorders and administration site conditions | Left hip and groin | Left hip and groin | 2:Moderate Adverse Event | Adverse Event Unrelated | Adverse Event Related | |  | No | No | Dose not changed |
| 1010006 | 1/Cohort 1 | Epistaxis | Respiratory, thoracic and mediastinal disorders |  |  | 2:Moderate Adverse Event | Adverse Event Related | Adverse Event Related | |  | Yes | No | Dose reduced |
| 1010007 | 1/Cohort 1 | Hypotension | Vascular disorders |  |  | 1:Mild Adverse Event | Adverse Event Unrelated | Adverse Event Unrelated | Adverse Event Related | Dehydration | No | No | Dose not changed |
| 1010007 | 1/Cohort 1 | Oral hemorrhage | Gastrointestinal disorders |  |  | 1:Mild Adverse Event | Adverse Event Related | Adverse Event Unrelated | Adverse Event Related | Recent dental work | Yes | No | Dose not changed |
| 1010007 | 1/Cohort 1 | Anemia | Blood and lymphatic system disorders |  |  | 2:Moderate Adverse Event | Adverse Event Related | Adverse Event Related | |  | No | No | Dose interrupted |
| 1010007 | 1/Cohort 1 | Tinnitus | Ear and labyrinth disorders |  |  | 1:Mild Adverse Event | Adverse Event Unrelated | Adverse Event Unrelated | Adverse Event Related | Ear congestion, allergic reaction | No | No | Dose not changed |
| 1010007 | 1/Cohort 1 | Hypopituitarism | Endocrine disorders |  |  | 2:Moderate Adverse Event | Adverse Event Unrelated | Adverse Event Unrelated | Adverse Event Related | Secondary Hypothyroidism | No | No | Dose not changed |
| 1010007 | 1/Cohort 1 | Back pain | Musculoskeletal and connective tissue disorders |  |  | 2:Moderate Adverse Event | Adverse Event Unrelated | Adverse Event Unrelated | Adverse Event Related | Laminectomy 8/4/23 | No | No | Dose interrupted |
| 1010007 | 1/Cohort 1 | Arthralgia | Musculoskeletal and connective tissue disorders | Intermittent Right leg pain |  | 1:Mild Adverse Event | Adverse Event Related | Adverse Event Unrelated | |  | No | No | Dose not changed |
| 1010007 | 1/Cohort 1 | Musculoskeletal and connective tissue disorder | Musculoskeletal and connective tissue disorders | Carpal tunnel syndrome (right hand/wrist) | Carpal tunnel syndrome | 2:Moderate Adverse Event | Adverse Event Unrelated | Adverse Event Unrelated | Adverse Event Related | Overuse of hand/wrist and typing | No | No | Dose not changed |
| 1010007 | 1/Cohort 1 | Arthralgia | Musculoskeletal and connective tissue disorders | Bilateral shoulders |  | 1:Mild Adverse Event | Adverse Event Related | Adverse Event Unrelated | |  | No | No | Dose not changed |
| 1010007 | 1/Cohort 1 | Arthralgia | Musculoskeletal and connective tissue disorders | Hands, wrists, shoulders, arms |  | 2:Moderate Adverse Event | Adverse Event Related | Adverse Event Unrelated | |  | No | No | Dose interrupted |
| 1010007 | 1/Cohort 1 | Arthralgia | Musculoskeletal and connective tissue disorders | Intermittent Right hand |  | 2:Moderate Adverse Event | Adverse Event Related | Adverse Event Unrelated | |  | No | No | Dose not changed |
| 1010008 | 2/Cohort 2 | Anemia | Blood and lymphatic system disorders | Shortness of break, weakness, swelling |  | 3:Severe Adverse Event | Adverse Event Unrelated | Adverse Event Related | Adverse Event Related | Prior radiation therapy | No | No | Dose interrupted |
| 1010008 | 2/Cohort 2 | Pleural effusion | Respiratory, thoracic and mediastinal disorders | Bilateral |  | 3:Severe Adverse Event | Adverse Event Unrelated | Adverse Event Related | |  | No | Yes | Dose interrupted |
| 1010008 | 2/Cohort 2 | Anorexia | Metabolism and nutrition disorders |  |  | 1:Mild Adverse Event | Adverse Event Unrelated | Adverse Event Related | |  | No | No | Dose not changed |
| 1010008 | 2/Cohort 2 | Pleural effusion | Respiratory, thoracic and mediastinal disorders | Seen on Chest XRAY |  | 1:Mild Adverse Event | Adverse Event Unrelated | Adverse Event Related | |  | No | No | Dose not changed |
| 1010008 | 2/Cohort 2 | Localized edema | General disorders and administration site conditions | Left arm, bilateral legs |  | 1:Mild Adverse Event | Adverse Event Unrelated | Adverse Event Related | |  | No | No | Dose not changed |
| 1010008 | 2/Cohort 2 | Fatigue | General disorders and administration site conditions | Includes weakness and exertional dyspnea |  | 3:Severe Adverse Event | Adverse Event Unrelated | Adverse Event Related | |  | No | No | Dose not changed |
| 1010008 | 2/Cohort 2 | Pleural effusion | Respiratory, thoracic and mediastinal disorders |  |  | 3:Severe Adverse Event | Adverse Event Unrelated | Adverse Event Related | |  | No | No | Dose not changed |
| 1010008 | 2/Cohort 2 | Insomnia | Psychiatric disorders |  |  | 1:Mild Adverse Event | Adverse Event Unrelated | Adverse Event Related | Adverse Event Related | Pain | No | No | Dose not changed |
| 1010008 | 2/Cohort 2 | Weight loss | Investigations |  |  | 1:Mild Adverse Event | Adverse Event Unrelated | Adverse Event Related | |  | No | No | Dose not changed |
| 1010009 | 1/Cohort 1 | Hyperhidrosis | Skin and subcutaneous tissue disorders |  |  | 1:Mild Adverse Event | Adverse Event Related | Adverse Event Related | |  | No | No | Dose not changed |
| 1010009 | 1/Cohort 1 | Constipation | Gastrointestinal disorders |  |  | 1:Mild Adverse Event | Adverse Event Unrelated | Adverse Event Related | Adverse Event Related | Traveling | No | No | Dose not changed |
| 1010009 | 1/Cohort 1 | Eye pain | Eye disorders |  |  | 1:Mild Adverse Event | Adverse Event Unrelated | Adverse Event Unrelated | Adverse Event Related | Dryness | No | No | Dose not changed |
| 1010009 | 1/Cohort 1 | Fatigue | General disorders and administration site conditions |  |  | 2:Moderate Adverse Event | Adverse Event Unrelated | Adverse Event Related | Adverse Event Related | Prior treatment | No | No | Dose not changed |
| 1010009 | 1/Cohort 1 | Constipation | Gastrointestinal disorders |  |  | 1:Mild Adverse Event | Adverse Event Related | Adverse Event Unrelated | |  | No | No | Dose not changed |
| 1010009 | 1/Cohort 1 | Upper respiratory infection | Infections and infestations |  |  | 2:Moderate Adverse Event | Adverse Event Unrelated | Adverse Event Unrelated | Adverse Event Related | Travel; Fatigue | No | No | Dose not changed |
| 1010009 | 1/Cohort 1 | Pruritus | Skin and subcutaneous tissue disorders |  |  | 1:Mild Adverse Event | Adverse Event Related | Adverse Event Unrelated | |  | No | No | Dose not changed |
| 1010010 | 2/Cohort 2 | Diarrhea | Gastrointestinal disorders |  |  | 1:Mild Adverse Event | Adverse Event Related | Adverse Event Unrelated | |  | No | No | Dose not changed |
| 1010010 | 2/Cohort 2 | Diarrhea | Gastrointestinal disorders |  |  | 2:Moderate Adverse Event | Adverse Event Related | Adverse Event Related | Adverse Event Related | COVID Booster | No | No | Dose not changed |
| 1010010 | 2/Cohort 2 | Platelet count decreased | Investigations |  |  | 3:Severe Adverse Event | Adverse Event Unrelated | Adverse Event Unrelated | Adverse Event Related | COVID Booster | No | No | Dose not changed |
| 1010010 | 2/Cohort 2 | Epistaxis | Respiratory, thoracic and mediastinal disorders |  |  | 3:Severe Adverse Event | Adverse Event Related | Adverse Event Related | |  | Yes | No | Dose not changed |
| 1010010 | 2/Cohort 2 | Acute kidney injury | Renal and urinary disorders |  |  | 3:Severe Adverse Event | Adverse Event Unrelated | Adverse Event Unrelated | Adverse Event Related | COVID Booster | No | No | Dose not changed |
| 1010010 | 2/Cohort 2 | Lipase increased | Investigations |  |  | 3:Severe Adverse Event | Adverse Event Unrelated | Adverse Event Unrelated | Adverse Event Related | COVID Booster | No | No | Dose not changed |
| 1010010 | 2/Cohort 2 | Hypokalemia | Metabolism and nutrition disorders |  |  | 1:Mild Adverse Event | Adverse Event Unrelated | Adverse Event Unrelated | Adverse Event Related | Diarrhea | No | No | Dose not changed |
| 1010010 | 2/Cohort 2 | Hypomagnesemia | Metabolism and nutrition disorders |  |  | 1:Mild Adverse Event | Adverse Event Unrelated | Adverse Event Unrelated | Adverse Event Related | Diarrhea | No | No | Dose not changed |
| 1010010 | 2/Cohort 2 | Oral hemorrhage | Gastrointestinal disorders |  |  | 1:Mild Adverse Event | Adverse Event Related | Adverse Event Unrelated | |  | Yes | No | Dose not changed |
| 1010010 | 2/Cohort 2 | Anemia | Blood and lymphatic system disorders |  |  | 2:Moderate Adverse Event | Adverse Event Related | Adverse Event Unrelated | |  | No | No | Dose not changed |
| 1010010 | 2/Cohort 2 | Neoplasms benign, malignant and unspecified | Neoplasms benign, malignant and unspecified | Keratocanthoma; Chest; Back of neck; Face; Arms | Keratoacanthoma | 1:Mild Adverse Event | Adverse Event Related | Adverse Event Unrelated | |  | No | No | Dose not changed |
| 1010010 | 2/Cohort 2 | Anemia | Blood and lymphatic system disorders |  |  | 2:Moderate Adverse Event | Adverse Event Related | Adverse Event Unrelated | |  | No | No | Dose not changed |
| 1010010 | 2/Cohort 2 | Pruritus | Skin and subcutaneous tissue disorders |  |  | 1:Mild Adverse Event | Adverse Event Related | Adverse Event Unrelated | |  | No | No | Dose not changed |
| 1010010 | 2/Cohort 2 | Back pain | Musculoskeletal and connective tissue disorders |  |  | 1:Mild Adverse Event | Adverse Event Unrelated | Adverse Event Unrelated | Adverse Event Related | Aging; Physical activity | No | No | Dose not changed |
| 1010010 | 2/Cohort 2 | Anemia | Blood and lymphatic system disorders | Headache, fatigue, syncope |  | 3:Severe Adverse Event | Adverse Event Related | Adverse Event Related | |  | No | No | Dose interrupted |
| 1010010 | 2/Cohort 2 | Lipase increased | Investigations |  |  | 2:Moderate Adverse Event | Adverse Event Related | Adverse Event Related | |  | No | No | Dose interrupted |
| 1010010 | 2/Cohort 2 | Sepsis | Infections and infestations | Fever, abdominal pain, sweats and chills, fatigue, elevated procalcitonin |  | 3:Severe Adverse Event | Adverse Event Unrelated | Adverse Event Unrelated | Adverse Event Related | COVID Booster 10/7 | No | Yes | Dose not changed |
| 1010011 | 1/Cohort 1 | Paresthesia | Nervous system disorders | Perianal |  | 1:Mild Adverse Event | Adverse Event Unrelated | Adverse Event Related | |  | No | No | Dose not changed |
| 1010011 | 1/Cohort 1 | Dysphasia | Nervous system disorders |  |  | 2:Moderate Adverse Event | Adverse Event Unrelated | Adverse Event Related | |  | No | No | Dose not changed |

**Supplementary Table 3.** All treatment-emergent AEs of any grade, any attribution. Duplicate AEs for individual patients represent separate instances of the same AE.

| **Patient** | **Date** | **Event** | **Days from treatment start** | **SLD** | **SLD change (%)** | **Response** | **Comment** |
| --- | --- | --- | --- | --- | --- | --- | --- |
| 1010001 | 2022-06-21 | Treatment start | 0.0 | 4.5 | 0.0 |  |  |
| 1010001 | 2022-08-01 | imaging | 41.0 | 4.6 | 2.2 | SD |  |
| 1010002 | 2022-09-22 | Treatment start | 0.0 | 2.0 | 0.0 |  |  |
| 1010002 | 2022-11-28 | imaging | 67.0 | 2.0 | 0.0 | SD |  |
| 1010002 | 2023-01-10 | imaging | 110.0 | 2.3 | 15.0 | SD |  |
| 1010002 | 2023-03-07 | imaging | 166.0 | 2.3 | 15.0 | SD |  |
| 1010002 | 2023-05-02 | imaging | 222.0 | 2.2 | 10.0 | SD |  |
| 1010002 | 2023-06-27 | imaging | 278.0 | 2.0 | 0.0 | SD |  |
| 1010002 | 2023-08-22 | imaging | 334.0 | 2.1 | 5.0 | SD |  |
| 1010002 | 2023-10-17 | imaging | 390.0 | 2.3 | 15.0 | SD |  |
| 1010002 | 2023-12-18 | imaging | 452.0 | 2.3 | 15.0 | PD | Non-target |
| 1010003 | 2022-10-11 | Treatment start | 0.0 | 4.8 | 0.0 |  |  |
| 1010003 | 2022-12-06 | imaging | 56.0 | 5.0 | 4.2 | SD |  |
| 1010003 | 2023-01-17 | imaging | 98.0 | 5.3 | 10.4 | SD |  |
| 1010003 | 2023-03-14 | imaging | 154.0 | 4.7 | -2.1 | SD |  |
| 1010003 | 2023-05-09 | imaging | 210.0 | 4.6 | -4.2 | SD |  |
| 1010003 | 2023-08-29 | imaging | 322.0 | 4.5 | -6.3 | SD |  |
| 1010003 | 2023-10-24 | imaging | 378.0 | 4.6 | -4.2 | SD |  |
| 1010003 | 2023-12-18 | imaging | 433.0 | 4.7 | -2.1 | SD |  |
| 1010003 | 2024-02-12 | imaging | 489.0 | 4.6 | -4.2 | SD |  |
| 1010003 | 2024-04-16 | imaging | 553.0 | 4.4 | -8.3 | SD |  |
| 1010004 | 2022-10-25 | Treatment start | 0.0 | 4.3 | 0.0 |  |  |
| 1010004 | 2022-12-19 | imaging | 55.0 | 4.6 | 6.9 | SD |  |
| 1010004 | 2023-02-13 | imaging | 111.0 | 5.4 | 25.6 | PD |  |
| 1010004 | 2023-03-27 | imaging | 153.0 | 5.8 | 34.9 | PD |  |
| 1010005 | 2022-12-06 | Treatment start | 0.0 | 5.3 | 0.0 |  |  |
| 1010005 | 2023-01-30 | imaging | 55.0 | 5.8 | 9.4 | SD |  |
| 1010005 | 2023-03-27 | imaging | 111.0 | 5.8 | 9.4 | PD | Non-target |
| 1010006 | 2023-02-07 | Treatment start | 0.0 | 1.9 | 0.0 |  |  |
| 1010006 | 2023-04-03 | imaging | 55.0 | 1.6 | -15.8 | SD |  |
| 1010006 | 2023-05-30 | imaging | 112.0 | 1.7 | -10.5 | SD |  |
| 1010006 | 2023-07-25 | imaging | 168.0 | 1.9 | 0.0 | SD |  |
| 1010006 | 2023-09-18 | imaging | 223.0 | 2.0 | 5.3 | SD |  |
| 1010007 | 2023-02-07 | Treatment start | 0.0 | 5.5 | 0.0 |  |  |
| 1010007 | 2023-04-03 | imaging | 55.0 | 5.5 | 0.0 | SD |  |
| 1010007 | 2023-05-30 | imaging | 112.0 | 5.6 | 1.8 | PD | Non-target |
| 1010008 | 2023-02-28 | Treatment start | 0.0 | 9.6 | 0.0 |  |  |
| 1010008 | 2023-03-28 | imaging | 28.0 |  |  | PD | Non-target |
| 1010009 | 2023-05-09 | Treatment start | 0.0 | 6.2 | 0.0 |  |  |
| 1010009 | 2023-07-03 | imaging | 55.0 | 6.6 | 6.5 | PD | Non-target |
| 1010009 | 2023-08-08 | imaging | 91.0 | 7.0 | 12.9 | PD |  |
| 1010010 | 2023-05-17 | Treatment start | 0.0 | 1.8 | 0.0 |  |  |
| 1010010 | 2023-07-11 | imaging | 55.0 | 1.7 | -5.6 | SD |  |
| 1010010 | 2023-09-12 | imaging | 118.0 | 1.5 | -16.7 | SD |  |
| 1010010 | 2023-10-18 |  | 154.0 |  |  | PD | Non-target |

**Supplementary Table 4.** RECIST measurements and BOR.

| **Patient** | **Collected Date Time** | **Observation Name** | **Observation Value** | **Observation Comment** | **Unit of Measure** | **Normal Range** |
| --- | --- | --- | --- | --- | --- | --- |
| 1010003 | 2024-03-18 | Neuron Specific Enolase | 14 |  | ng/mL | <=15 |
| 1010003 | 2024-03-18 | Chromogranin A | 281 | H | ng/mL | <93 |
| 1010003 | 2024-02-12 | Neuron Specific Enolase | 9.8 |  | ng/mL | <=15 |
| 1010003 | 2024-02-12 | Chromogranin A | 358 | H | ng/mL | <93 |
| 1010003 | 2023-12-18 | Neuron Specific Enolase | 9.9 |  | ng/mL | <=15 |
| 1010003 | 2023-12-18 | Chromogranin A | 320 | H | ng/mL | <93 |
| 1010003 | 2023-10-23 | Neuron Specific Enolase | 11 |  | ng/mL | <=15 |
| 1010003 | 2023-10-23 | Chromogranin A | 334 | H | ng/mL | <93 |
| 1010003 | 2023-09-26 | Neuron Specific Enolase | 11 |  | ng/mL | <=15 |
| 1010003 | 2023-09-26 | Chromogranin A | 397 | H | ng/mL | <93 |
| 1010003 | 2023-08-28 | Neuron Specific Enolase | 9.4 |  | ng/mL | <=15 |
| 1010003 | 2023-08-28 | Chromogranin A | 389 | H | ng/mL | <93 |
| 1010003 | 2023-07-03 | Neuron Specific Enolase | 8.2 |  | ng/mL | <=15 |
| 1010003 | 2023-07-03 | Chromogranin A | 255 | H | ng/mL | <93 |
| 1010003 | 2023-05-08 | Neuron Specific Enolase | 9.8 |  | ng/mL | <=15 |
| 1010003 | 2023-05-08 | Chromogranin A | 280 | H | ng/mL | <93 |
| 1010003 | 2023-03-13 | Neuron Specific Enolase | 8.1 |  | ng/mL | <=15 |
| 1010003 | 2023-03-13 | Chromogranin A | 290 | H | ng/mL | <93 |
| 1010003 | 2023-01-17 | Neuron Specific Enolase | 11 |  | ng/mL | <=15 |
| 1010003 | 2023-01-17 | Chromogranin A | 318 | H | ng/mL | <93 |
| 1010003 | 2022-12-05 | Chromogranin A | 395 | H | ng/mL | <93 |
| 1010003 | 2022-12-05 | Neuron Specific Enolase | 11 |  | ng/mL | <=15 |
| 1010003 | 2022-10-25 | Chromogranin A | 273 | H | ng/mL | <93 |
| 1010003 | 2022-10-25 | Neuron Specific Enolase | 11 |  | ng/mL | <=15 |
| 1010011 | 2023-06-12 | Neuron Specific Enolase | 12 |  | ng/mL | <=15 |
| 1010011 | 2023-06-12 | Chromogranin A | 106 | H | ng/mL | <93 |
| 1010010 | 2023-09-11 | Neuron Specific Enolase | 51 | H | ng/mL | <=15 |
| 1010010 | 2023-09-11 | Chromogranin A | 834 | H | ng/mL | <93 |
| 1010010 | 2023-07-10 | Neuron Specific Enolase | 34 | H | ng/mL | <=15 |
| 1010010 | 2023-07-10 | Chromogranin A | 624 | H | ng/mL | <93 |
| 1010010 | 2023-05-30 | Neuron Specific Enolase | 22 | H | ng/mL | <=15 |
| 1010010 | 2023-05-17 | Chromogranin A | 344 | H | ng/mL | <93 |
| 1010005 | 2024-03-04 | Neuron Specific Enolase | 14 |  | ng/mL | <=15 |
| 1010005 | 2024-03-04 | Chromogranin A | 69 |  | ng/mL | <93 |
| 1010005 | 2023-03-27 | Neuron Specific Enolase | 19 | H | ng/mL | <=15 |
| 1010005 | 2023-03-27 | Chromogranin A | 158 | H | ng/mL | <93 |
| 1010005 | 2023-01-30 | Neuron Specific Enolase | 16 | H | ng/mL | <=15 |
| 1010005 | 2023-01-30 | Chromogranin A | 201 | H | ng/mL | <93 |
| 1010005 | 2022-11-28 | Chromogranin A | 93 | H | ng/mL | <93 |
| 1010005 | 2022-11-28 | Neuron Specific Enolase | 24 | H | ng/mL | <=15 |
| 1010004 | 2023-03-27 | Neuron Specific Enolase | 29 | H | ng/mL | <=15 |
| 1010004 | 2023-03-27 | Chromogranin A | 203 | H | ng/mL | <93 |
| 1010004 | 2023-02-13 | Neuron Specific Enolase | 16 | H | ng/mL | <=15 |
| 1010004 | 2023-02-13 | Chromogranin A | 254 | H | ng/mL | <93 |
| 1010004 | 2022-12-19 | Chromogranin A | 207 | H | ng/mL | <93 |
| 1010004 | 2022-12-19 | Neuron Specific Enolase | 12 |  | ng/mL | <=15 |
| 1010004 | 2022-10-21 | Chromogranin A | 162 | H | ng/mL | <93 |
| 1010004 | 2022-10-21 | Neuron Specific Enolase | 11 |  | ng/mL | <=15 |
| 1010006 | 2024-03-18 | Neuron Specific Enolase | 11 |  | ng/mL | <=15 |
| 1010006 | 2024-03-18 | Chromogranin A | 93 | H | ng/mL | <93 |
| 1010006 | 2023-12-11 | Neuron Specific Enolase | 9.5 |  | ng/mL | <=15 |
| 1010006 | 2023-12-11 | Chromogranin A | 98 | H | ng/mL | <93 |
| 1010006 | 2023-09-18 | Neuron Specific Enolase | 12 |  | ng/mL | <=15 |
| 1010006 | 2023-09-18 | Chromogranin A | 63 |  | ng/mL | <93 |
| 1010006 | 2023-07-24 | Neuron Specific Enolase | 8.9 |  | ng/mL | <=15 |
| 1010006 | 2023-07-24 | Chromogranin A | 60 |  | ng/mL | <93 |
| 1010006 | 2023-05-30 | Neuron Specific Enolase | 13 |  | ng/mL | <=15 |
| 1010006 | 2023-05-30 | Chromogranin A | 51 |  | ng/mL | <93 |
| 1010006 | 2023-04-03 | Neuron Specific Enolase | 10 |  | ng/mL | <=15 |
| 1010006 | 2023-04-03 | Chromogranin A | 52 |  | ng/mL | <93 |
| 1010006 | 2023-02-07 | Neuron Specific Enolase | 11 |  | ng/mL | <=15 |
| 1010006 | 2023-02-07 | Chromogranin A | 46 |  | ng/mL | <93 |
| 1010006 | 2023-01-10 | Chromogranin A | 48 |  | ng/mL | <93 |
| 1010006 | 2023-01-10 | Neuron Specific Enolase | 12 |  | ng/mL | <=15 |
| 1010002 | 2024-02-12 | Neuron Specific Enolase | 13 |  | ng/mL | <=15 |
| 1010002 | 2024-02-12 | Chromogranin A | 134 | H | ng/mL | <93 |
| 1010002 | 2024-01-16 | Neuron Specific Enolase | 12 |  | ng/mL | <=15 |
| 1010002 | 2024-01-16 | Chromogranin A | 90 |  | ng/mL | <93 |
| 1010002 | 2023-12-18 | Neuron Specific Enolase | 12 |  | ng/mL | <=15 |
| 1010002 | 2023-12-18 | Chromogranin A | 133 | H | ng/mL | <93 |
| 1010002 | 2023-11-14 | Neuron Specific Enolase | 13 |  | ng/mL | <=15 |
| 1010002 | 2023-11-14 | Chromogranin A | 124 | H | ng/mL | <93 |
| 1010002 | 2023-10-16 | Neuron Specific Enolase | 11 |  | ng/mL | <=15 |
| 1010002 | 2023-10-16 | Chromogranin A | 118 | H | ng/mL | <93 |
| 1010002 | 2023-09-19 | Neuron Specific Enolase | 12 |  | ng/mL | <=15 |
| 1010002 | 2023-09-19 | Chromogranin A | 151 | H | ng/mL | <93 |
| 1010002 | 2023-08-21 | Neuron Specific Enolase | 13 |  | ng/mL | <=15 |
| 1010002 | 2023-08-21 | Chromogranin A | 171 | H | ng/mL | <93 |
| 1010002 | 2023-06-26 | Neuron Specific Enolase | 11 |  | ng/mL | <=15 |
| 1010002 | 2023-06-26 | Chromogranin A | 96 | H | ng/mL | <93 |
| 1010002 | 2023-06-13 | Neuron Specific Enolase | 11 |  | ng/mL | <=15 |
| 1010002 | 2023-06-13 | Chromogranin A | 102 | H | ng/mL | <93 |
| 1010002 | 2023-05-01 | Neuron Specific Enolase | 11 |  | ng/mL | <=15 |
| 1010002 | 2023-05-01 | Chromogranin A | 127 | H | ng/mL | <93 |
| 1010002 | 2023-03-06 | Neuron Specific Enolase | : |  | NA |  |
| 1010002 | 2023-03-06 | Chromogranin A | 98 | H | ng/mL | <93 |
| 1010002 | 2023-02-21 | Neuron Specific Enolase | 11 |  | ng/mL | <=15 |
| 1010002 | 2023-02-21 | Chromogranin A | 92 |  | ng/mL | <93 |
| 1010002 | 2023-02-07 | Neuron Specific Enolase | 11 |  | ng/mL | <=15 |
| 1010002 | 2023-02-07 | Chromogranin A | 139 | H | ng/mL | <93 |
| 1010002 | 2023-01-09 | Chromogranin A | 126 | H | ng/mL | <93 |
| 1010002 | 2023-01-09 | Neuron Specific Enolase | 11 |  | ng/mL | <=15 |
| 1010002 | 2022-11-28 | Chromogranin A | 93 | H | ng/mL | <93 |
| 1010002 | 2022-11-28 | Neuron Specific Enolase | 8.7 |  | ng/mL | <=15 |
| 1010002 | 2022-09-20 | Chromogranin A | 82 |  | ng/mL | <93 |
| 1010002 | 2022-09-20 | Neuron Specific Enolase | 11 |  | ng/mL | <=15 |
| 1010007 | 2023-11-13 | Neuron Specific Enolase | 39 | H | ng/mL | <=15 |
| 1010007 | 2023-11-13 | Chromogranin A | 694 | H | ng/mL | <93 |
| 1010007 | 2023-05-30 | Neuron Specific Enolase | 46 | H | ng/mL | <=15 |
| 1010007 | 2023-05-30 | Chromogranin A | 1117 | H | ng/mL | <93 |
| 1010007 | 2023-04-18 | Neuron Specific Enolase | 35 | H | ng/mL | <=15 |
| 1010007 | 2023-04-03 | Chromogranin A | 650 | H | ng/mL | <93 |
| 1010007 | 2023-02-07 | Neuron Specific Enolase | 30 | H | ng/mL | <=15 |
| 1010007 | 2023-02-07 | Chromogranin A | 493 | H | ng/mL | <93 |
| 1010009 | 2023-08-07 | Chromogranin A | 231 | H | ng/mL | <93 |
| 1010009 | 2023-07-03 | Neuron Specific Enolase | 116 | H | ng/mL | <=15 |
| 1010009 | 2023-07-03 | Chromogranin A | 215 | H | ng/mL | <93 |
| 1010009 | 2023-05-23 | Neuron Specific Enolase | 68 | H | ng/mL | <=15 |
| 1010009 | 2023-05-23 | Chromogranin A | 122 | H | ng/mL | <93 |
| 1010009 | 2023-05-09 | Neuron Specific Enolase | 53 | H | ng/mL | <=15 |
| 1010009 | 2023-05-09 | Chromogranin A | 98 | H | ng/mL | <93 |

**Supplementary Table 5.** Longitudinal serum neuron specific enolase and chromogranin A values.

**SUPPLEMENTARY MATERIAL**

**Trial Participation Eligibility Criteria**

Inclusion criteria:

1. Histologically or cytologically confirmed recurrent or metastatic ONB not amenable to potentially curative local therapies. Review of tissue samples by Pathology at the NIH is preferred.
2. Participants must have measurable disease, per RECIST 1.1. See Section 6.3 for the evaluation of measurable disease. A previously treated lesion by radiotherapy can be chosen as the target lesion only if progression in the respective lesion has been demonstrated during or following radiotherapy.
3. Participants should have received at least one line of systemic therapy including a platinum agent, with evidence of disease progression clinically or radiographically.
4. Men or Women; Age >18 years.
5. ECOG performance status ≤2.
6. Adequate hematologic function at screening, as follows:
   a. Absolute neutrophil count (ANC) ≥1500/mcL;
   b. Hemoglobin ≥ 9 g/dL;
   c. Platelets ≥ 100,000/microliter.
7. Adequate renal and hepatic function at screening, as follows:
   a. Serum creatinine ≤ 1.5 x upper limit of normal (ULN) OR Measured or calculated creatinine clearance/eGFR ≥ 30 mL/min for participant with creatinine levels > 1.5 X institutional ULN (eGFR by CKD-EPI formula);
   b. Total Bilirubin ≤ 1.5 x ULN OR direct ≤ ULN if total bilirubin > 1.5 x ULN;
   c. Alanine aminotransferase (ALT) and aspartate aminotransferase (AST) ≤ 2.5 x ULN.
8. The effects of immunotherapy on the developing human fetus are unknown. Therefore, participants must use effective methods of contraception (such as implants, injectables, combined oral contraceptives, IUDs, sexual abstinence or vasectomized partner).
   a. Women of child-bearing potential (WOCBP: any woman who has experienced menarche and has not had hysterectomy or bilateral oophorectomy or is not postmenopausal (amenorrheic 12 months or more following cessation of exogenous hormonal treatments; if <50 years old need follicle stimulating hormone FSH in the post-menopausal range)) must agree to use highly effective contraception prior to study entry and for up to 65 days following the last dose of study treatment.
   b. Men must agree to use highly effective contraception prior to study entry and up to 125 days following the last dose of study treatment.
   c. Should a woman become pregnant or suspect she is pregnant while she or her partner is participating in this study, she should inform her treating physician immediately.
9. Participants with bone metastases or hypercalcemia on intravenous bisphosphonate treatment, zolendronic acid, denosumab, or similar agents are eligible to participate and may continue this treatment.
10. Participants with treated CNS ONB lesions are eligible if follow-up brain imaging after at least a month following central nervous system (CNS)-directed therapy shows no evidence of progression.
11. Participants with new or progressive non-intraparenchymal CNS ONB lesions are eligible if the treating physician determines that immediate CNS specific treatment is not required and is unlikely to be required during the first cycle of therapy.
12. Human immunodeficiency virus (HIV)-positive participants must have CD4 count ≥ 200 cells per cubic millimeter at enrollment, be on stable antiretroviral therapy for at least 4 weeks and have no reported opportunistic infections or Castleman’s disease within 12 months prior to enrollment.
13. For participants with serological evidence of chronic hepatitis B virus (HBV) infection, the HBV DNA viral load must be undetectable on suppressive therapy, if indicated.
14. For participants with serological evidence of HCV infection, the HCV RNA viral load must be negative to be eligible for study participation.
15. Ability of participant to understand and the willingness to sign a written informed consent document.
16. Must co-enroll in the following two studies. A separate inform consent will be obtained from participant for these studies.
    a. 21-C-0009: “A Natural History Study of Children and Adults with Olfactory Neuroblastoma”
    b. 18-DC-0051: “Biospecimen procurement for NIDCD clinical protocols”

**Exclusion Criteria**

1. Anticancer treatment, concurrent or prior (chemotherapy, monoclonal antibody, cytokine therapy, immune therapy, targeted small molecule therapy) or any investigational drug, within 4 weeks or 5 half-lives (whichever shorter) prior to the first drug administration. All residual treatment-related toxicities must have resolved or be minimal and not constitute a safety risk. Note: Palliative radiotherapy is permitted concurrently or within the pretreatment period. Subjects receiving bisphosphonates or denosumab are eligible provided treatment was initiated at least 14 days before treatment.
2. Participants who received prior checkpoint blockade therapy and were taken off treatment for serious adverse events related to immuno-therapy are excluded.
3. Major surgery within 4 weeks prior to the first drug administration (minimally invasive procedures such as diagnostic biopsies are permitted).
4. Active or prior documented autoimmune or inflammatory diseases that might deteriorate on immunostimulatory agent (including colitis or Crohn’s disease, systemic lupus erythematosus, sarcoidosis, vasculitis, Grave’s disease, hypophysitis, uveitis, rheumatoid arthritis etc.), except the following:
   a. Type I diabetes mellitus
   b. Chronic skin conditions that do not require systemic therapy (including eczema, vitiligo, alopecia, psoriasis)
   c. Hypothyroidism (e.g., post-Hashimoto thyroiditis) stable, on hormone replacement
   d. Mild autoimmune disease not active in the last 5 years may be eligible after consultation with the principal investigator.
5. Current use of immunosuppressive medication within 14 days before the first dose of the study medication, except the following:
   a. Intranasal, inhaled, topical glucocorticoids; locally injected glucocorticoids (i.e. intra-articular, intra-ocular)
   b. Systemic glucocorticoids at physiologic doses (generally ≤ 10 mg prednisone or equivalent per day)
   c. Glucocorticoids as premedication for contrast-enhanced studies is allowed prior to enrollment and on study.
6. Uncontrolled intercurrent chronic or acute illness including, but not limited to the following, that may limit interpretation of results or increase risk to the participant in the judgment of the investigator:
   a. Bleeding diathesis or recent (<3 months) clinically significant bleeding event.
   b. Prior organ transplantation including allogenic stem-cell transplantation
   c. Impaired cardiovascular function or clinically significant cardiovascular disease, including, but not limited to, any of the following:
      i. cerebral vascular accident/stroke (< 3 months prior to enrollment),
      ii. acute coronary syndromes (including myocardial infarction < 6 months prior to enrollment, unstable angina),
      iii. Congestive Heart Failure (≥ New York Heart Association Classification Class III); CHF Class II must have been stable for 3 months prior to enrollment
      iv. history or presence of clinically significant cardiac arrhythmia including resting bradycardia, uncontrolled atrial fibrillation or paroxysmal supraventricular tachycardia (controlled arrhythmias, e.g. stable atrial fibrillation, may be allowed at the discretion of the investigator),
      v. history of myocarditis
   d. History of idiopathic pulmonary fibrosis, drug-induced or idiopathic pneumonitis, active interstitial lung disease, blood oxygen saturation <90% at rest (on ambient air).
   e. Clinically significant hepatic disease.
   f. Active infection requiring systemic therapy (minor infections may be allowed at the discretion of the investigator).
7. Subjects unwilling to accept blood products as medically indicated.
8. Vaccination with live vaccines within 4 weeks of the first dose of treatment and while on study is prohibited. Inactivated vaccines may be administered.
9. History of allergic reactions or hypersensitivity attributed to compounds of similar chemical or biologic composition to bintrafusp alfa. Participants with history of severe hypersensitivity reaction to monoclonal antibodies (grade ≥ 3 NCI-CTCAE v5) will be evaluated by the allergy/immunology team prior to enrollment.
10. History of second malignancy within 3 years of enrollment except for the following: adequately treated localized basal cell or squamous skin cancer, cervical carcinoma in situ, superficial bladder cancer, other localized malignancy which has been adequately treated or malignancy which does not require active systemic treatment (e.g. low risk CLL).
11. Pregnant or breastfeeding women are excluded from this study because the study medications have not been tested in pregnant women and there is potential for teratogenic or abortifacient effects. Because there is an unknown but potential risk for adverse events in nursing infants secondary to treatment of the mother with the study medications, breastfeeding should be discontinued if the mother is treated on this protocol.
